# Computational Study of Protein-Ligand Unbinding for Enzyme Engineering

**DOI:** 10.3389/fchem.2018.00650

**Published:** 2019-01-08

**Authors:** Sérgio M. Marques, David Bednar, Jiri Damborsky

**Affiliations:** ^1^Loschmidt Laboratories, Department of Experimental Biology and Research Centre for Toxic Compounds in the Environment RECETOX, Faculty of Science, Masaryk University, Brno, Czechia; ^2^International Clinical Research Center, St. Anne's University Hospital Brno, Brno, Czechia

**Keywords:** unbinding kinetics, protein engineering, molecular dynamics, metadynamics, adaptive sampling, CaverDock

## Abstract

The computational prediction of unbinding rate constants is presently an emerging topic in drug design. However, the importance of predicting kinetic rates is not restricted to pharmaceutical applications. Many biotechnologically relevant enzymes have their efficiency limited by the binding of the substrates or the release of products. While aiming at improving the ability of our model enzyme haloalkane dehalogenase DhaA to degrade the persistent anthropogenic pollutant 1,2,3-trichloropropane (TCP), the DhaA31 mutant was discovered. This variant had a 32-fold improvement of the catalytic rate toward TCP, but the catalysis became rate-limited by the release of the 2,3-dichloropropan-1-ol (DCP) product from its buried active site. Here we present a computational study to estimate the unbinding rates of the products from DhaA and DhaA31. The metadynamics and adaptive sampling methods were used to predict the relative order of kinetic rates in the different systems, while the absolute values depended significantly on the conditions used (method, force field, and water model). Free energy calculations provided the energetic landscape of the unbinding process. A detailed analysis of the structural and energetic bottlenecks allowed the identification of the residues playing a key role during the release of DCP from DhaA31 via the main access tunnel. Some of these hot-spots could also be identified by the fast CaverDock tool for predicting the transport of ligands through tunnels. Targeting those hot-spots by mutagenesis should improve the unbinding rates of the DCP product and the overall catalytic efficiency with TCP.

## Introduction

Until recently, the modern methods of structure-based drug design relied primarily on the high binding affinity toward the targets to predict their biological performance. However, that paradigm has been changed once it was realized that the half-life of a drug is equally important to define its *in vivo* efficacy, and hence both thermodynamics and kinetics profiles must be taken into account (Lu and Tonge, 2010). For this reason, we have recently witnessed a boom of different methods for the computational prediction of receptor-ligand (un)binding kinetics (Chiu and Xie, 2016; Ferruz and De Fabritiis, 2016; Dickson et al., 2017; Rydzewski and Nowak, 2017; Bruce et al., 2018; Kokh et al., 2018). The importance of determining association and dissociation rates (*k*_on_ and *k*_off_, respectively), however, is not restricted to the field of drug design. In structural biology and biocatalysis, the study of the thermodynamics and kinetics of binding and unbinding can be very important to attain a deep understanding of the biological processes of interest. There are well-known cases where the substrate binding or the product release are the rate-limiting steps in the catalytic cycle (Wang et al., 2001; Bosma et al., 2003; Yao et al., 2005). Interestingly, it has been shown that the substrate unbinding, under certain condition, may also have a positive impact on the enzymatic turnover (Reuveni et al., 2014). Therefore, the computational study of the (un)binding processes might reveal their kinetic and/or thermodynamics bottlenecks, and, in some cases, lead to finding improved biocatalysts for biotechnological applications.

**Scheme 1:**
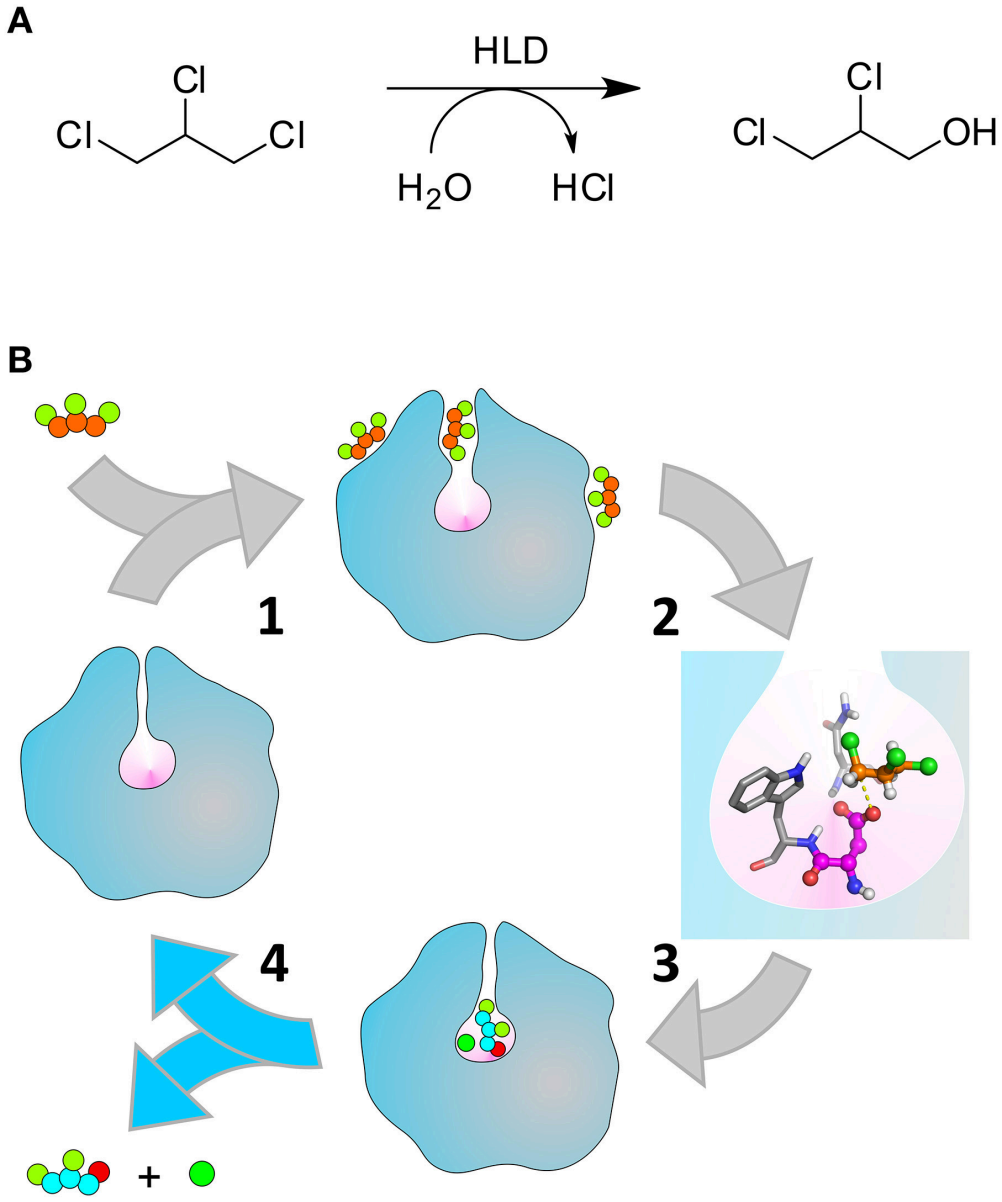
Catalytic cycle of the HLDs. **(A)** Hydrolytic dehalogenation reaction catalyzed by the HLDs converting TCP to DCP. **(B)** Illustration of the respective catalytic cycle: (1) binding of TCP (orange and green balls) to the enzyme tunnels that connect the buried active site (pink cavity) to the bulk solvent; (2) formation of the pre-reactive Michaelis complex (the nucleophile D106 and the halide-stabilizing residues N41-W107 are represented in magenta and gray, respectively), (3) chemical steps converting TCP into DCP (cyan, green, and red balls) and Cl^−^ (dark green ball), (4) release of the products to regenerate the free enzyme. The unbinding of DCP (step 4, highlighted with the blue arrows) is the kinetic bottleneck that limits the rate of DhaA31, and is the focus of this work. Adapted with permission from Marques et al. (2017). Copyright 2017 American Chemical Society.

**Figure 1:**
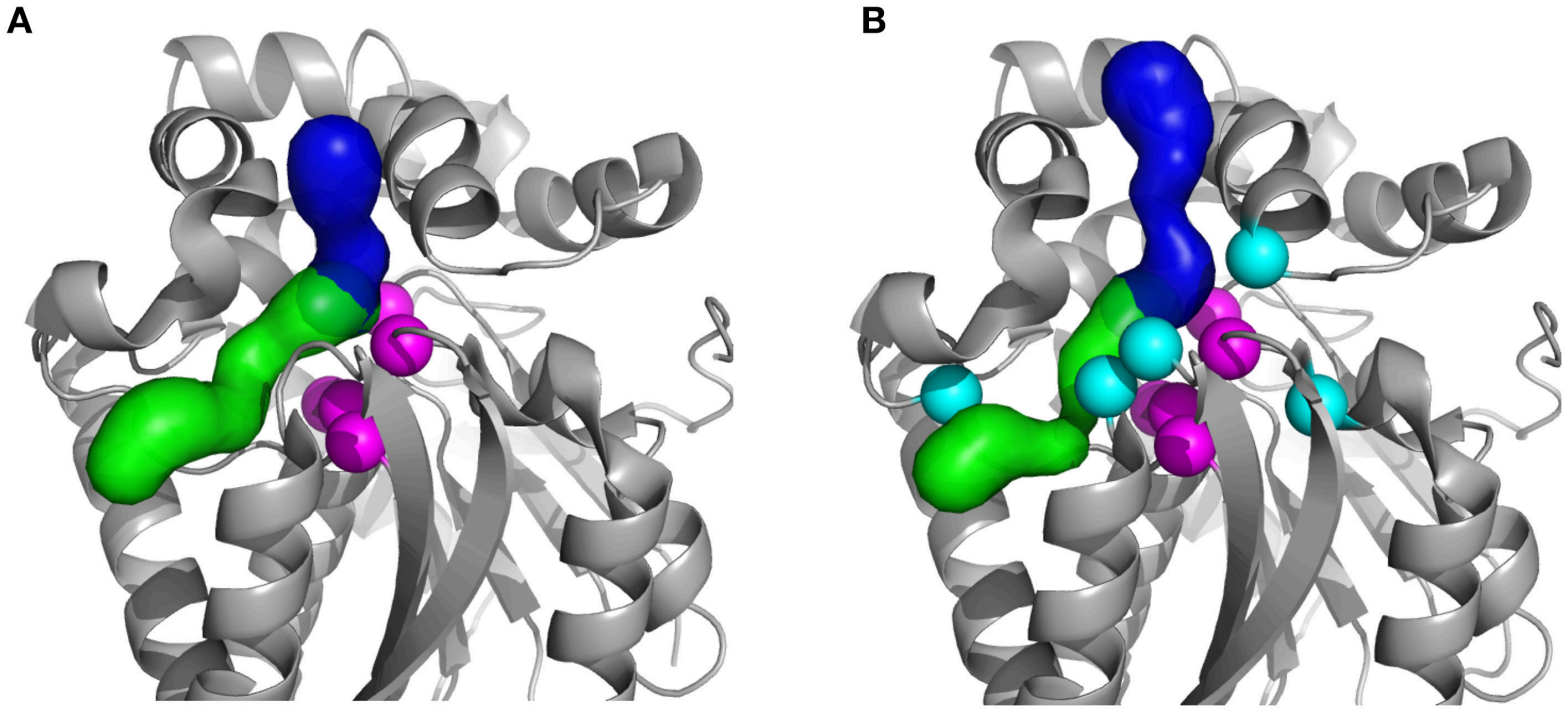
Structures of the studied HLDs with their respective tunnels. **(A)** Crystal structures of the wild-type DhaA (DhaAwt; PDB-ID: 4E46) and **(B)** DhaA31 mutant (PDB-ID: 3RK4). The tunnels were calculated using CAVER 3.02 (Chovancova et al., 2012): the main tunnel (p1) is shown as the blue surface and the slot tunnel (p2) as green; the catalytic residues (C_α_ atoms) are represented as magenta spheres and the mutations in DhaA31 (I135F, C176Y, V245F, L246I, and Y273F) as cyan spheres.

The haloalkane dehalogenases (HLDs, E.C.3.8.1.5) are one of such cases. These bacterial enzymes can perform the hydrolytic conversion of halogenated aliphatic compounds into the respective alcohols (Scheme 1A). They have several practical applications, namely in the synthesis of enantiopure chemical compounds, recycling of by-products, bioremediation, and biosensing (Koudelakova et al., 2013). As several other haloalkanes, 1,2,3-trichloropropane (TCP) is an anthropogenic compound which sometimes ends up contaminating the groundwater as a recalcitrant toxic pollutant. Therefore, biodegradation would be a possible solution for the remediation of the contaminated sites (Samin and Janssen, 2012). The HLD from *R. rhodochrous*, DhaA, can only moderately hydrolyze TCP into 2,3-dichloropropan-1-ol (DCP). However, the 5-point mutant DhaA31 (Figure 1) has been reported to display a turnover number enhanced by 32-fold, resulting in a turnover number (*k*_cat_) of 1.26 s^−1^ (Pavlova et al., 2009). DhaA31 is currently one of the best known HLDs in hydrolyzing TCP, and it has been included in the biodegradation pathway to stepwise convert the toxic TCP into glycerol (Dvorak et al., 2014; Kurumbang et al., 2014).

The HLDs have a buried active site connected to the surface by molecular tunnels (Figure 1). Their catalytic cycle (Scheme 1B) consists of: the substrate binding to the enzyme (1), rearrangement of the substrate in the catalytic site to form a reactive configuration (2), a multi-stage chemical step (3), and the release of the alcohol and halide products to regenerate the free enzyme (4). The chemical step involves an S_N_2 attack of the D106 nucleophile on the electrophilic carbon atom of the substrate (DhaA numeration according to UniProt ID P0A3G2). The halide ion and the alkyl-enzyme intermediate are formed, the latter is attacked by a water molecule, activated by the catalytic base H272, which ultimately leads to the final products (Verschueren et al., 1993; Kutý et al., 1998; Marques et al., 2017). It is known that the hydrolysis of TCP by the wild-type DhaA (DhaAwt) is rate-limited by the S_N_2 reaction, while in DhaA31 the slowest step is the release of DCP. This knowledge was attained from comparison of steady-state kinetic rates with pre-steady-state rates (Pavlova et al., 2009; Marques et al., 2017). Moreover, the mutations C176F and V245F in DhaA31 contributed the most to the improvement of the S_N_2 step toward TCP, whilst most of its bulky mutations—including C176F—narrow down the molecular tunnels and thus hinder the release of the alcohol product (Marques et al., 2017).

By accelerating the DCP unbinding from DhaA31 with mutagenesis, without hampering other steps in the catalytic cycle, we might improve the efficiency of DhaA31 to degrade TCP even further, which is desirable for biotechnological applications. We have recently targeted the geometric bottleneck of DhaA31's main tunnel, with mutations introduced to position 176, that had a high impact on the catalysis with different substrates (Kaushik et al., 2018). However, only minor improvements in the activity toward TCP were attained. The present work allows us to tackle the challenge from a new perspective.

Here we report a thorough computational study of the unbinding of DCP from DhaA31 and DhaAwt. Initially, we calculated the kinetic rates using metadynamics (MTD), and adaptive sampling under different simulation conditions. This helped us to assess the best procedures for predicting absolute and relative unbinding rates. Next, we performed free energy calculations using funnel-metadynamics (funnel-MTD), and calculated the energetic profiles of the product unbinding. This allowed us to compare the energy barriers, identify the thermodynamic bottlenecks, and thus predict several hot-spots for mutagenesis that could potentially improve the release of the DCP product and thus enhance the conversion of TCP by DhaA31.

## Materials and Methods

### Metadynamics Unbinding Kinetics

#### System Preparation

The complexes of DhaA31 and DhaAwt bound with DCP and chloride products in their active site were prepared using the positions of DCP (only the R-enantiomer was studied here) docked into the corresponding crystal structures (PDB entries 3RK4 and 4E46, respectively), protonated and treated as previously described (Marques et al., 2017). The positions of the Cl^−^ ion were taken from the respective crystal structures. The PREPI parameters for DCP were prepared using the Antechamber module of AmberTools 14 (Case et al., 2014), from the MOL2 structure containing the partial atomic charges, as previously calculated (Marques et al., 2017), and compiled using the atom types of GAFF force field. The topology and coordinates of the complexes hydrated only with the crystallographic water molecules were generated with tLEaP module of AmberTools 14, with the protein, and ions described by the AMBER ff12SB force field (Maier et al., 2015), and converted to the GROMACS format using the ACPYPE script (Sousa da Silva and Vranken, 2012). Each system was solvated with a cubic box of TIP3P water molecules (Jorgensen et al., 1983) with the edges at least 8 Å away from the protein atoms and then neutralized with Na^+^ ions using the *editconf* module of GROMACS 5.0 package (Abraham et al., 2015).

#### System Equilibration

Energy minimization was performed with GROMACS 5.0.7 (Abraham et al., 2015) without restraints to relax the whole system, using the steepest descent method until the maximum force converged to values below 1 kJ/mol·nm with a maximum of 500 steps. The Particle Mesh Ewald method was used for the treatment of the long range non-bonding interactions beyond the 10 Å cut-off (Darden et al., 1993), and the periodic boundary condition was applied. Equilibration dynamics was run in two steps: a first equilibration of 500 ps in the isothermal-isobaric ensemble (*NPT*), at 1 atm, with the isotropic Berendsen barostat (Berendsen et al., 1984), and coupling constant 0.2 ps, and a second one of 1 ns in the isothermal-isochoric ensemble (*NVT*). Both steps were conducted at 300 K with the velocity-rescaled Berendsen thermostat, to ensure the proper canonical ensemble (Bussi et al., 2007), with constant for coupling of 0.1 ps. All simulations were performed with the periodic boundary conditions in all directions, the Verlet pair-lists scheme (Verlet, 1967) with cut-off values of 10 Å for both short-range coulombic and van der Waals potentials, and the LINear Constraint Solver (LINCS) (Hess et al., 1997) algorithm to constrain the bonds and eliminate drifts. The integration time step was 2 fs and the energy and coordinates of the system were recorded every 1 ps.

#### Setup of the Collective Variable

A path-based collective variable (path CV) was defined to describe the release process of DCP along the p1 tunnel, according to the formalism as previously described (Branduardi et al., 2007; Bonomi et al., 2008). It involves a distance *s* along a reference path that leads from state A (the fully bound state, the docked conformation in the active site) to B (fully unbound state, with DCP in the bulk solvent). The path was constructed based on several snapshots selected from previous accelerated molecular dynamics (aMD) simulations with DhaA31 and DhaAwt (Marques et al., 2017) to have DCP at different distances and orientations between states A and B. In total 9 frames were chosen for each system, and only the ligand and the residues of p1 tunnel in contact with DCP during the release were selected as the path reference (Figure S1 and Table S1). The path CV (hereafter termed *p3*) was then defined by the root mean square deviation (RMSD) space. From a further analysis of a set of unbinding metadynamics simulations, it was found that the direct variable of the path *s* (named *p3.sss*) was degenerated and hence was not suitable to be used alone in this study. The degeneracy was lifted using a second CV, which was the distance (*d1*) between the center of mass of DCP to the active site cavity, defined by the center of mass of the atoms Y176-C_β_, F205-C_α_, L209-C_α_, and H272-C_α_ for DhaA31, and C176-C_β_, F205-C_α_, L209-C_α_, and H272-C_α_ for DhaAwt. The λ parameter was set to 92 for the DhaA31/DCP and 100 for DhaAwt/DCP. The values of λ were obtained from the analysis of the RMSD matrix obtained from the frames.

#### Infrequent Metadynamics Simulations

All metadynamics (MTD) simulations were performed using PLUMED (Tribello et al., 2014) plugin, version 2.2.3 with the GROMACS 5.0.7 (Abraham et al., 2015) code. The *NVT* ensemble at 300 K was used as in the equilibration, with further position restraints on the atoms Leu36-C_α_, Ile104-C_α_ and Leu237-C_α_ with harmonic constant of 2.38 kcal/mol·Å^2^ (1,000 kJ/mol·nm^2^) in each dimension, to prevent drifting of the protein across the periodic cell. The potential biases were added to the path CV *s* dimension and the distance *d* variables, deposited every 50 ps, with initial height of 0.60 kcal/mol (2.50 kJ/mol) for both variables. The Gaussian widths (σ) for *s* and *d1* were set, respectively, as 0.05 and 0.014 Å for DhaA31/DCP and 0.07 and 0.013 Å for DhaAwt/DCP, and a decay corresponding to a bias factor of 10. In total 25 independent infrequent MTD simulations were run until the ligand was released to reach distances *d1* > 22 Å from the active site without immediate rebinding. These times corresponded to the biased release times, *t*_*biased*_. The trajectories were visualized using VMD 1.9.1 (Humphrey et al., 1996) and PyMOL 1.7.4 (The PyMO L Molecular Graphics System, 2014).

To obtain the unbiased release time *t*_*unbiased*_, the acceleration factor α was used as describe by Equations 1, 2 (Tiwary and Parrinello, 2013; Tiwary et al., 2015):

(1) $$\begin{array}{rcl} \alpha & = & \langle e^{\frac{V\left(r,t\right)}{k_{B}T}}\rangle \end{array}$$

(2) $$\begin{array}{rcl} t_{unbiased} & = & t_{biased}\times \alpha \end{array}$$

where 〈 〉 denotes the running average accumulated through the course of the simulation at the biased time, *t* or *t*_*biased*_, *V*(*r*,*t*) is the time-dependent metadynamics bias, *r* the set of CV descriptors, and *k*_B_*T* is the temperature in energy units, which has the value of 2.50 kJ·mol^−1^ at 300 K.

#### Calculation of k_off_ From Metadynamics

The estimation of the unbinding rates *k*_off_ involves the calculation of the characteristic transition time τ of a Poisson process, obtained through a least squares fitting of the empirical cumulative distribution function (ECDF) obtained with the metadynamics unbiased times, *t*_*unbiased*_, with the theoretical expression of a cumulative distribution function (TCDF), which in the case of a homogeneous Poisson process is given by Equation (3) (Tiwary et al., 2015):

(3) $$\begin{array}{r} \text{TCDF}=1-e^{-\frac{t}{\tau }} \end{array}$$

The theoretical (TCDF) and empirical (ECDF) distributions are compared by a Kolmogorov-Smirnoff test, which estimates an associated *p-value*, which represents the probability that the distribution of times extracted from metadynamics is obtained from the theoretical exponential distribution, and describes the quality of the data (Salvalaglio et al., 2014; Tiwary et al., 2015). Acceptable distributions should always present *p-value* >0.05, otherwise the set of results is discarded. To perform the fitting of those distributions, the Kolmogorov-Smirnov test, and calculate the dissociation transition time τ_off_, the ***STPtest.m*** Matlab script was used as provided (Salvalaglio et al., 2014). The dissociation rate *k*_off_ was then calculated from τ_off_ by the Equation 4:

(4) $$\begin{array}{r} k_{off}=\frac{1}{\tau_{off}} \end{array}$$

The error associated with the calculated *k*_off_ value was estimated by a bootstrap analysis of the data set of unbiased release times obtained for each system. This was performed by re-analyzing 500 sub-samples extracted randomly from the original ensemble of release times.

### Adaptive Sampling Kinetics

#### System Preparation

The complexes of DhaA31 and DhaAwt bound with DCP and chloride products in their active site, hydrated with the crystallographic waters, were prepared as described for the metadynamics. Na^+^ and Cl^−^ ions were added in order to achieve ionic strength of 0.1 M, and a TIP3P (Jorgensen et al., 1983) cubic box of water molecules with the edges 10 Å distant from the original system was added. The topology and coordinates of the hydrated complexes were generated with tLEaP module of AmberTools 14 (Case et al., 2014), with the protein and ions described with the ff12SB AMBER force field (Hornak et al., 2006; Joung and Cheatham, 2008, 2009; Nguyen et al., 2014). For comparison testing different simulation conditions, the systems were also prepared with force field ff14SB (Maier et al., 2015) and OPC3 water model (Izadi and Onufriev, 2016).

#### System Equilibration

The systems were equilibrated using the *Equilibration_v2* module of high-throughput molecular dynamics (HTMD) (Doerr et al., 2016). The system was first minimized using conjugate-gradient method for 500 steps. Then the system was heated and minimized as follows: (I) 500 steps (2 ps) of *NVT* equilibration with the Berendsen barostat to 298 K, with constraints on all heavy atoms of the protein, (II) 625 000 steps (2.5 ns) of NPT equilibration with Langevin thermostat with 1 kcal·mol^−1^·Å^−2^ constraints on all heavy atoms of the protein, and (III) 625 000 steps (2.5 ns) of NPT equilibration with the Langevin thermostat without constraints. During the equilibration simulations, holonomic constraints were applied on all hydrogen-heavy atom bond terms and the mass of the hydrogen atoms was scaled with factor 4, enabling the simulations to run with 4 fs time steps (Feenstra et al., 1999; Harvey and De Fabritiis, 2009; Harvey et al., 2009; Hopkins et al., 2015). The simulations employed periodic boundary conditions, using the particle mesh Ewald method for treatment of interactions beyond 9 Å cut-off, electrostatic interactions suppressed for more than 4 bond terms away from each other and the smoothing and switching van der Waals and electrostatic interaction cut-off at 7.5 Å (Harvey and De Fabritiis, 2009).

#### Adaptive Sampling

The HTMD was used to perform adaptive sampling of the RMSD of the Cα atoms. The 20 ns production molecular dynamics (MD) runs were started with the system resulting from the equilibration cycle and employed the same settings as the last step of the equilibration. The trajectories were saved every 0.1 ns. Adaptive sampling was performed using the distance between the central C-2 atom of DCP and the Cγ atom of the catalytic nucleophile D106 as the reaction coordinate, and a time-based independent component analysis (TICA) (Naritomi and Fuchigami, 2011) in 1 dimension. Unless stated otherwise, 40 epochs of 10 MDs each were performed for DhaA31 and 30 epochs for DhaAwt, corresponding to cumulative simulation times of 8 and 6 μs, respectively.

#### Markov State Model Construction

The simulations were made into a simulation list using HTMD method and water was filtered out, and unsuccessful simulations with length <20 ns were omitted. This resulted in 8 μs of simulation time (400 × 20 ns) for DhaA31 and 6 μs of simulation time (300 × 20 ns) for DhaAwt (Doerr et al., 2016). The DCP dynamics was studied by the distance between the C-2 atom of DCP and the Cγ atom of the catalytic nucleophile D106. The data was clustered using MiniBatchKmeans algorithm to 200 clusters. 15 ns lag time was used in the models to construct 3 Markov states, and the Chapman-Kolmogorov test was performed to assess the quality of the constructed states. A bootstrapping calculation was performed with 80% of the data and repeated 500 times to estimate the errors in the estimated kinetic parameters.

### Funnel Metadynamics

The MTD simulations were performed using the GROMACS 5.0.7 (Abraham et al., 2015) patched with the PLUMED plugin (Tribello et al., 2014), version 2.2.3, modified to include the funnel metadynamics (funnel-MTD) algorithm and used as provided by the authors of the method (Limongelli et al., 2013). The *NVT* ensemble at 300 K was used as previously, with the further position restraints on the Leu36-C_α_, Ile104-C_α_, and Leu237-C_α_ atoms with a harmonic force constant of 59.8 kcal/mol·Å^2^ (25,000 kJ/mol·nm^2^) in each dimension to prevent the protein from drifting across the periodic cell. These atoms were chosen for being buried and having some of the lowest B-factors in the respective crystal structures. The potential biases were added to the path CV *s* variable, deposited every 1 ps, with initial height of 0.60 kcal/mol (2.50 kJ/mol). The Gaussian width (σ) was 0.05 Å for DhaA31/DCP and 0.07 and 0.013 Å for DhaAwt/DCP, as previously, and a decay corresponding to a bias factor of 10. A funnel-shaped restraint with 83.6 kcal/mol.A^2^ (35,000 kJ/mol.nm^2^) force constant, was defined by the *Z* axis passing through the points A—the coordinates of the D106-C_α_ atom—and B—the geometric center of the F144-C_α_, F152-C_α_, A167-C_α_, and K175-C_α_ atoms –, the α angle of 0.55 rad, *Z*_*cc*_ 20.0 Å, and *R*_*cyl*_ 5.0 Å. To prevent the ligand from crossing the periodic cell, an upper distance restraint with 12.0 kcal/mol.A^2^ (5,000 kJ/mol.nm^2^) force constant was imposed at 23 Å from point A. The free energy surface (FES) was computed using the SUM_HILLS module of PLUMED, from the histogram distribution reweighted from the biases added by the metadynamics (Bonomi et al., 2009; Tiwary and Parrinello, 2015). The FES was reanalyzed for the variable *d1*, defined above, using the DRIVER module of PLUMED. The histogram reweighting was performed by taking into account all the biases from the metadynamics and the restraints. The relevant states were selected from the FES, and the simulation snapshots corresponding to *d1* values within ± 0.01 Å were extracted using GROMACS 5.0.7 (Abraham et al., 2015), in PDB format, after the trajectory was aligned by the C_α_ atoms.

### Binding Free Energy

The free energy of binding was calculated by the molecular mechanics/generalized Born solvent accessible surface area (MM/GBSA) method to determine the interaction energy of DCP with the protein residues in each one of the ensembles obtained from the selected states of the FES. For that, the topology of the systems in the GROMACS format were converted to the AMBER format using the ParmEd program (Swails, 2010). The *ante-MMPBSA.py* (Miller et al., 2012) module of AmberTools 14 (Case et al., 2014) was used to remove the solvent and ions from the resulting topology files and define the Born radii as *mbondi2*, and generate the corresponding topologies of the *complex, receptor*, and *ligand*, to be used in the MM/GBSA calculations. The state ensembles, in PDB format, were manually stripped from any ions and solvent. The *MMPBSA.py.MPI* (Miller et al., 2012) module of AmberTools 14 was used to calculate, in parallel, the mean free energy of binding considering every snapshot of the PDB ensemble. The generalized Born method was used (&*gb* namelist) with implicit generalized Born solvent model (*igb*=*8*) and 0.1 M ionic strength (*saltcon*=*0.1*). Decomposition of the pairwise interactions were generated (&*decomp* namelist) with discrimination of all types of energy contributions (*idecomp*=*4*) for the whole residue (*dec_verbose*=*0*).

### CaverDock Simulations

#### Tunnel Calculations

CAVER 3.02 (Chovancova et al., 2012) was used to calculate the tunnels in the protein structure of the DhaA31 and DhaAwt, as previously prepared and treated prior to the metadynamics. The tunnels were calculated using a probe radius of 0.7 Å, a shell radius of 3 Å, and shell depth 4 Å. The starting point for the tunnel calculation was the same point in the active site as previously used to calculate the distance *d1* (center of mass of the atoms Y176-C_β_, F205-C_α_, L209-C_α_, and H272-C_α_ for DhaA31, and C176-C_β_, F205-C_α_, L209-C_α_, and H272-C_α_ for DhaAwt).

#### CaverDock Calculations

CaverDock package (“CaverDock,” 2018; Filipovič et al., 2018) was used to calculate the trajectories of DCP through the p1 tunnel of DhaA31 and DhaAwt, as calculated by CAVER. The input files previously prepared for the receptors and ligand, respectively, in PDB or MOL2 format, were converted to the AutoDock Vina-compatible PDBQT format using the MGLTools v1-5-7rc1 (Morris et al., 2009), preserving the previously calculated partial charges for the ligand. The tunnels were extended by 6 Å and discretized with 0.3 Å increments. The ligand started in the active site and it was moved toward the protein surface. The side chain flexibility was iteratively introduced using the default settings, with two automatically chosen tunnel residues made flexible per iteration.

## Results

### Metadynamics Unbinding Kinetics

The unbinding of the alcohol product has become the rate-limiting step in the catalytic conversion of TCP by DhaA31, and that step is expected to be slower than with the wild-type DhaAwt (Pavlova et al., 2009; Marques et al., 2017). Aiming at verifying this computationally, we have used a state-of-the-art method for the calculation of the kinetic rates of ligand unbinding, which is based on metadynamics (Tiwary et al., 2015).

The metadynamics (MTD) relies on a set of collective variables (CVs) to describe the system and the process under study. Here we have made use of the knowledge acquired from a previous computational work (Marques et al., 2017) to define the unbinding path of DCP. In that study, the release of DCP from the active site was observed with both DhaA31 and DhaAwt, always through the main tunnel (p1 tunnel; see Figure 1), and these simulations were used to provide several frames for describing the path CV for those two systems. A discussion on the optimization of the CV is presented in Discussion S1 in Supplementary Material.

**Figure 2:**
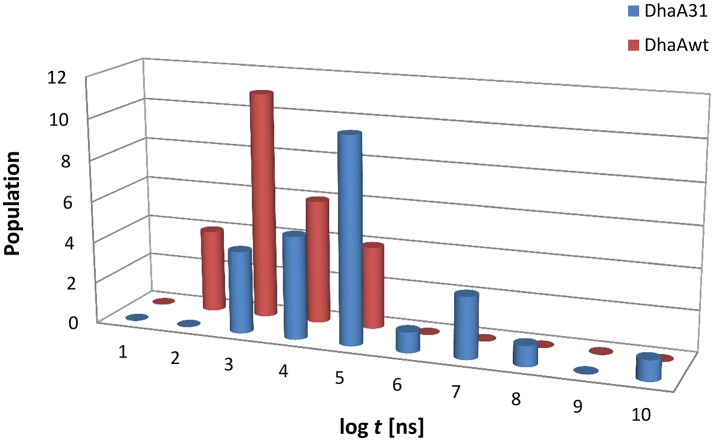
Histogram distribution of the unbiased release times (log *t*) of DCP from the buried active sites of **(A)** DhaA31 and **(B)** DhaAwt. The release times were obtained from the infrequent metadynamics simulations (25 runs were performed for each system).

Twenty five MTD simulations were performed with each protein containing DCP in their active site, and run until DCP was fully released to the bulk solvent (distance *d1* > 22 Å from the active site) without immediate rebinding to the tunnel. The release times obtained from the MTD simulations were converted to unbiased times, and the results showed a large dispersion of the release times for each system. But, as expected, in average DhaAwt released DCP significantly faster than DhaA31 (Figure 2 and Figure S2). The unbiased release times were fitted to determine the distribution and probabilities of the transitions (the ligand unbinding; see Figure S3). These analyses resulted in *p-values* above the minimum confidence threshold of 0.05 (Table S2), and the average dissociation times (τ_off_) and kinetic constants (*k*_off_) were obtained (Table 1). The evolution analysis of the τ_off_ with the number of simulation runs showed that the estimation of the dissociation time was well-converged even using lower number of simulations (Figure S4). With transition times in the range of microsecond timescales, the predicted *k*_off_ value was 36-fold faster for DhaAwt than for DhaA31 (Table 1). Such trend is in agreement with the previous findings that DCP was significantly more prone to be released from DhaAwt than from DhaA31 (Marques et al., 2017).

**Table 1:** Experimental and theoretical kinetic parameters obtained from the metadynamics and adaptive sampling simulations of DCP with DhaA31 and DhaAwt for all the tested force fields and water models.

	**Parameter^a^**	**Experimental**	**Metadynamics**	**Adaptive sampling**		
			**ff12SB+TIP3P^b^**	**ff12SB+TIP3P^b^**	**ff14SB+TIP3P^b^**	**ff14SB+OPC3^b^**
DhaA31	τ_off_ [ns]		3.5 ± 1.4 × 10^4^	188 ± 28	350 ± 49	2.2 ± 1.1 × 10^3^
	*k*_off_ [s^−1^]		2.8 ± 1.2 × 10^4^	5.32 ± 0.72 × 10^6^	2.86 ± 0.38 × 10^6^	4.5 ± 1.3 × 10^5^
	τ_on_ [ns]			1.74 ± 0.45 × 10^3^	5.1 ± 3.9 × 10^3^	5.7 ± 2.6 × 10^4^
	*k*_on_ [M^−1^s^−1^]			1.28 ± 0.34 × 10^8^	4.4 ± 1.4 × 10^7^	3.9 ± 1.5 × 10^6^
	*k*_off_/*k*_on_ [M]			0.042 ± 0.014	0.065 ± 0.053	0.116 ± 0.073
	*K*_d_ [M]	> 0.020^c^		0.057 ± 0.017	0.12 ± 0.10	0.77 ± 0.48
	ΔG${}_{\text{eq}}^{0}$ [kcal/mol]			−1.76 ± 0.19	−1.33 ± 0.3	−0.16 ± 0.33
DhaAwt	τ_off_ [ns]		9.9 ± 5.6 × 10^2^	75 ± 11	73.0 ± 5.3	163 ± 29
	*k*_off_ [s^−1^]	>>10^3^^d^	1.01 ± 0.56 × 10^6^	1.33 ± 0.18 × 10^7^	1.37 ± 0.09 × 10^7^	6.2 ± 1.2 × 10^6^
	τ_on_ [ns]			9.3 ± 7.1 × 10^3^	1.72 ± 0.54 × 10^3^	337 ± 127
	*k*_on_ [M^−1^s^−1^]			2.4 ± 1.4 × 10^7^	1.33 ± 0.27 × 10^8^	6.7 ± 2.9 × 10^8^
	*k*_off_/*k*_on_ [M]			0.54 ± 0.42	0.103 ± 0.035	9.1 ± 4.0 × 10^−3^
	*K*_d_ [M]	0.95 ± 0.34 × 10^−3^		0.42 ± 0.38	0.132 ± 0.030	8.3 ± 2.5 × 10^−3^
	ΔG${}_{\text{eq}}^{0}$ [kcal/mol]			−0.54 ± 0.34	−1.25 ± 0.13	−2.95 ± 0.18
DhaAwt/31	Rel. *k*_off_		36	2.5	4.8	14

a τ_off_, mean dissociation transition time; k_off_, dissociation rate; τ_on_, mean association transition time; k_on_, association rate; K_d_, equilibrium dissociation constant; ΔG${}_{\text{eq}}^{0}$, free energy difference between bound and unbound states; rel. k_off_, DhaAwt/DhaA31 ratio of k_off_ rates;

b force field and water model used;

c solubility concentration;

d *detection limit of the instrument. The variability of the parameters is the SD obtained from a bootstrap analysis*.

### Adaptive Sampling Kinetics

At this point we wanted to validate the kinetic rates previously calculated with the MDT approach by using another advanced and independent method. Thereby we can compare and assess the reproducibility of the kinetic predictions using different methods. So, we decided to perform high-throughput molecular dynamics (HTMD) using the adaptive sampling technique in combination with Markov state models (MSMs). This method allows us to obtain the transition matrix between the states and thus predict the kinetic rates of unbinding (Doerr et al., 2016).

**Figure 3:**
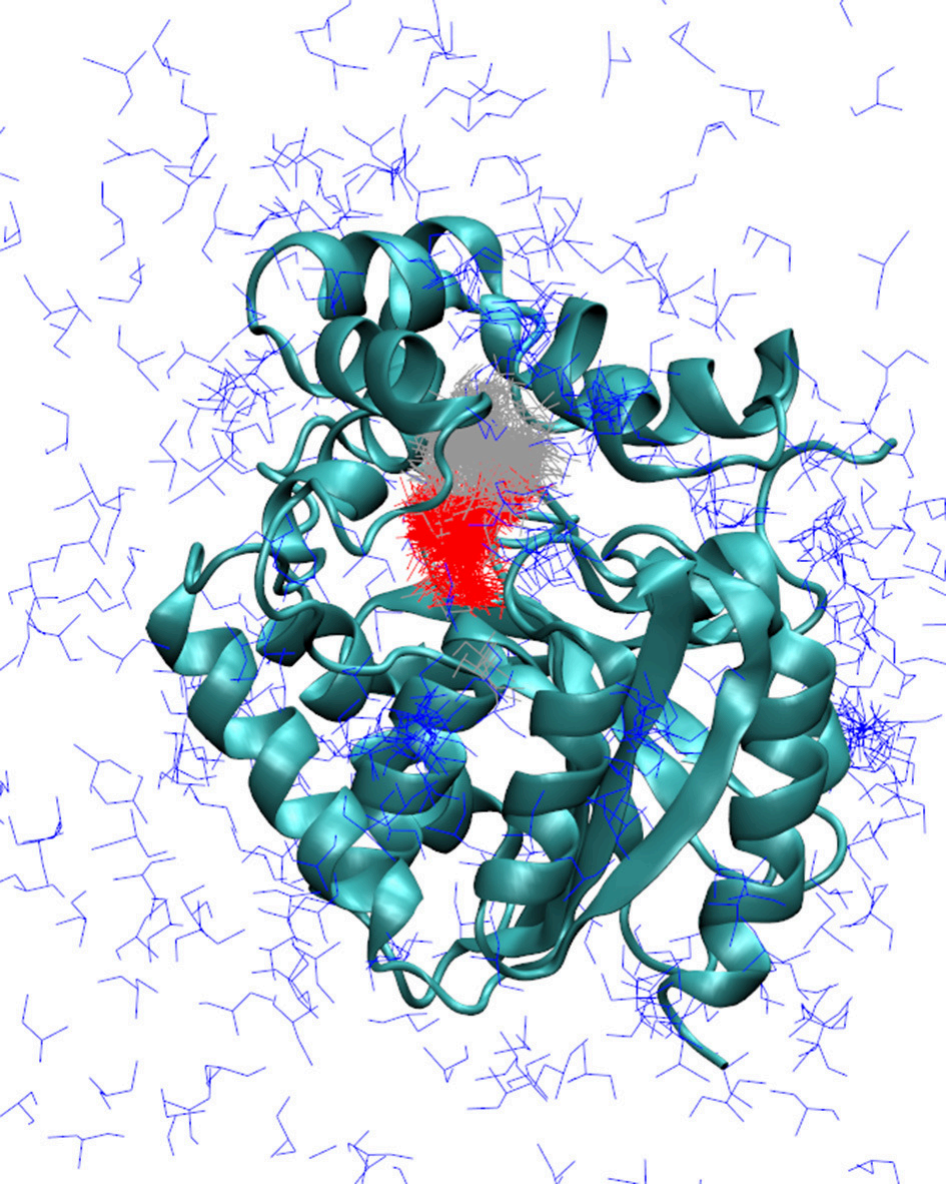
Markov state models describing the release of DCP in the simulations with adaptive sampling. Representative models of DCP with DhaA31: DCP fully bound in the active site (red), intermediate state with DCP bound in the access tunnel (gray), and unbound state (blue). The enzyme is represented as cyan cartoon. The Markov models obtained with DhaAwt were similar and are not shown here.

Initially, the adaptive simulations were performed using the same force field and water model as the MTD simulations (ff12SB and TIP3P). We found that the distance between the ligand and the catalytic nucleophile D106 (defined as described in the methods) was a good metrics for calculating the Markov state models for describing the events that we wanted to survey (the release of DCP from DhaA31 and DhaAwt). We defined 3 Markov states which were satisfying when visually inspected: one state corresponded to DCP located in the active site, an intermediate state with DCP in the main access tunnel (p1), and the unbound state with DCP outside the protein (Figure 3). The Chapman-Kolmogorov test was performed to assess the quality of the Markov state models, which revealed satisfying for the parameters used (Figures S5–S7). The kinetic rates between the fully bound and fully unbound states were calculated (Table 1). The default simulation time was 8 μs for DhaA31 and 6 μs for DhaAwt, which generally proved satisfactory according to the errors obtained by bootstrapping. The release rates of DCP (*k*_off_) obtained from the current adaptive sampling method (Table 1, column “ff12SB+TIP3P”) were 1-2 orders of magnitude higher than the ones previously calculated with the MTD method. Regarding the relative values of *k*_off_ values between DhaAwt and DhaA31, the order is maintained but with smaller difference between the two enzymes. DCP was released 2.5 times faster from DhaAwt than from DhaA31, which is only moderately in agreement with the MTD results (36 times faster for DhaAwt) and previous computational evidence (Marques et al., 2017). Regarding the remaining kinetic parameters, DhaA31 seemed to be more prone for rebinding DCP than DhaAwt, showing higher *k*_on_ and lower *K*_d_ values than DhaAwt. This is not in agreement with the experimental data, which showed higher *K*_d_ for DhaA31 than for DhaAwt (Table 1). The free energy of the bound state, compared to the unbound state, was −1.76 ± 0.19 kcal/mol for DhaA31, and −0.54 ± 0.34 kcal/mol for DhaAwt. This means that DCP's bound state in DhaA31 is thermodynamically more stable than that of DhaAwt by 1.22 kcal/mol. Furthermore, the proteins remained stable throughout the simulations, as can be inferred from the RMSD plots (Figure S8).

Due to the dissimilarity between the values and the kinetic rates' ratios obtained from MTD and adaptive sampling, we decided to perform a similar study under different simulation condition. For that, we varied the force field (ff14SB instead of ff12SB), and the water model (OPC3 instead of TIP3P). The *k*_off_ values obtained with ff14SB were lower than with ff12SB for both systems (Table 1), suggesting that the dynamic properties with ff14SB are slower than with ff12SB. When the OPC3 water model was used, slower unbinding rates were observed, as compared to those obtained with TIP3P. Other kinetic parameters were also significantly affected by the force field and solvent model, in some cases differing by orders of magnitude (namely *k*_on_, *K*_d_, and ΔG${}_{\text{eq}}^{0}$). The *K*_d_ values predicted with ff14SB+OPC3 approached the experimental ones more than the other conditions (8.3 ± 2.5 mM prediction vs. 0.95 mM experimental for DhaAwt). In some cases, the dynamic behavior of the systems changed so significantly that the initial simulation times (8 μs for DhaA31 and 6 μs for DhaAwt) did not provide enough sampling to produce precise estimations. This occurred for DhaA31 with ff14SB+OPC3 and for DhaAwt with ff14SB+TIP3P. In these cases, the simulations were run twice as long (16 μs for DhaA31 and 12 μs for DhaAwt), although for DhaA31 even a longer simulation time might be required due to the timescales of the observed events. Overall, the ff14SB+OPC3 combination seemed to produce more accurate results, with predicted *K*_d_ values closer to the experimental ones.

### Pre-steady State Kinetics

To validate the findings of the present computational study, the calculated kinetic properties were compared with the previously reported results from transient kinetic measurements.

The basis for the current work is that the rate-limiting step in the catalytic conversion of TCP by the DhaA31 mutant is the product release, i.e., unbinding of DCP from the protein to the bulk solvent. This conclusion has been made based on comparison of steady-state kinetic rates with results from the transient kinetics measurements (Pavlova et al., 2009). In this study we observed, that after a rapid mixing of DhaA31 with excess of TCP, there was a burst of both DCP and chloride, followed by a linear steady-state phase with the rate constant 1.36 ± 0.18 s^−1^ for DCP. This rate is in a very good agreement with the value from the steady-state kinetics *k*_cat_ 1.26 ± 0.07 s^−1^. Further studies showed that the release of the halide was a fast process,

(5) $$\begin{array}{r} k_{obs}=k_{1}+\frac{k_{-1}\;\cdot \;K_{d}}{\left[L\right]\;+\;K_{d}} \end{array}$$

which allowed us to conclude that the unbinding of DCP is rate-limiting for DhaA31, while DhaAwt is limited by the catalytic step (Marques et al., 2017).

**Scheme 2:**
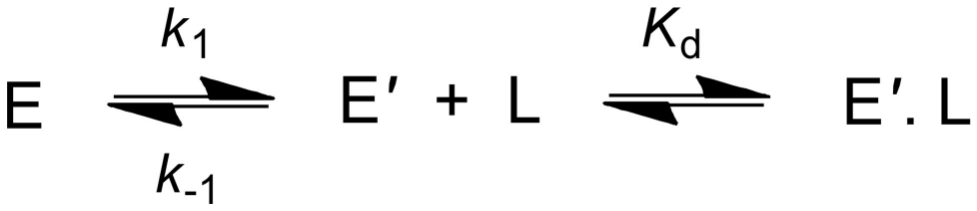
Kinetic scheme showing the binding of DCP (L) to the enzyme (E). The transition of E to E' represents a conformational change in the enzyme.

Binding experiments of DCP were also carried out with DhaA31 and DhaAwt using stopped-flow fluorescence. Unfortunately, these experiments proved unsuccessful with DhaA31 due to the very low affinity of this enzyme for DCP, for which no binding was observed at concentrations near the solubility limit. This also implies a high dissociation constant, with value *K*_d_ > 20 mM. For DhaAwt, the fluorescence curves revealed a slow kinetics profile upon mixing with DCP that could be associated with a single exponential. This was an indication that a slow conformational change of the enzyme preceded the fast binding of DCP (Scheme 2 and Equation 5). This fact, together with the time scale limitations of the instruments, disallowed the calculation of the binding, and unbinding rates of DCP, *k*_on_ and *k*_off_, but the equilibrium dissociation constant was obtained as *K*_d_ = 0.95 ± 0.34 mM. Moreover, the ratio between the enzyme conformations in equilibrium, E and E', favor the nonbinding form (E) by 2:1, with *k*_1_ = 3.31 ± 0.27 s^−1^ and *k*_−1_ = 6.16 ± 0.42 s^−1^ (Marques et al., 2017).

### Free Energy Calculations

The free energy profiles allow a deep understanding of the thermodynamic and kinetic determinants of individual steps of the catalytic cycle, since they reveal the energy of the different states and the energy barriers to the required transitions. Therefore, their study can be important to asses not only the differences between the unbinding of DCP from DhaA31 and DhaAwt, but also to understand how the current bottlenecks can be overcome in the scope of protein engineering.

The funnel-metadynamics (funnel-MTD) is a method that allows the efficient calculation of the free energy surface (FES) of the process of ligand (un)binding (Limongelli et al., 2013). In this method, a funnel-shaped restraint prevents the ligand from drifting away and thus allows sampling several forward and reverse events (unbinding/binding), needed for a correct estimation of the free energy profile associated with the process. The funnel restraints used in this study (Figure S9) were defined iteratively in order to allow the free motion of the ligand within the active site, main tunnel (p1), and respective tunnel mouth. The funnel-MTD simulations were performed using the same path CV as previously used in the MTD unbinding kinetics. The simulations were run until the free energy achieved convergence. In these simulations DCP was released (reaching distances *d1* > 15 Å) and rebound to the active site (reaching *d1* < 5 Å) several times with each system, as desirable for the free-energy calculations (Figure S10). In both cases, the proteins remained stable throughout most of the simulations, with only a steep increase in the RMSD of DhaAwt backbone between 100 and 110 ns, which was reversible to levels below 1.2 Å (Figure S11).

The free energy was primarily calculated with respect to the original CV used in the MTD simulations, and computed by reweighting the histogram distribution with the biasing Gaussians added to the system. this resulted in the respective one-dimensional FES (Bonomi et al., 2009; Tiwary and Parrinello, 2015). To analyze the convergence, the FES was calculated cumulatively by taking into account an increasing number of snapshots. Two energy basins were integrated and the difference between those basins was plotted as a function of the increasing simulation time (Figure S12). Similarly, the energy barrier between the same two energy basins was measured and plotted as a function of the simulation time (Figure S13). Altogether, this analysis allowed us to conclude that the simulations were well-converged after the respective running times (500 ns for DhaA31 and 400 ns for DhaAwt).

**Figure 4:**
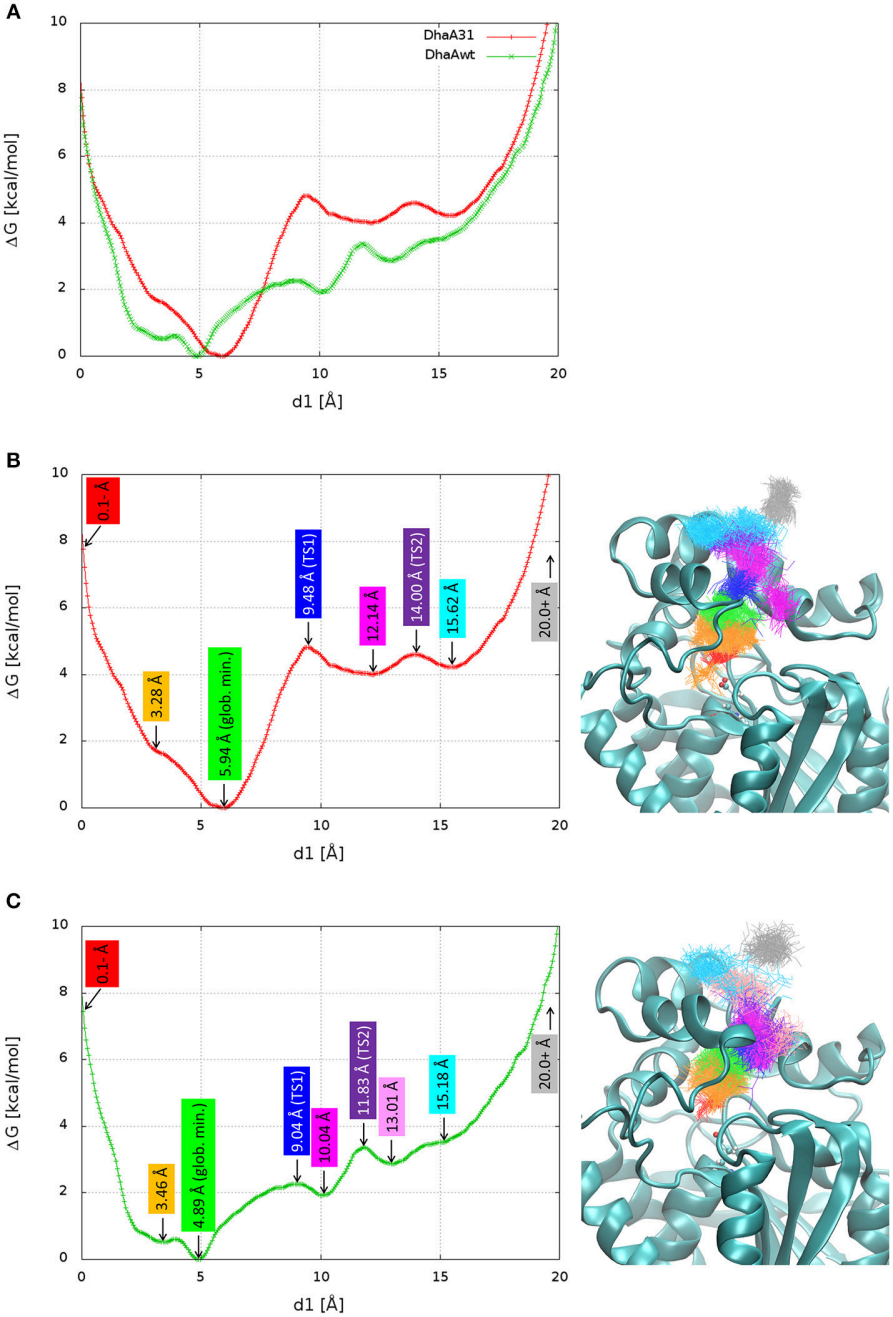
Free energy surface (FES) and structures of the relevant stages in the release of DCP. The FES projected on the distance of DCP to the active site (*d1*) superimposed for DhaA31 and DhaAwt **(A)**, and dissected with the relevant states for DhaA31 **(B)** and DhaAwt **(C)**. The relevant states are labeled with the corresponding *d1* values (left), and the respective clusters are shown by the superimposed DCP structures (right), represented as lines of the same colors. The protein is represented as cyan cartoons and the catalytic D106 as ball-and-sticks. Glob. min. global minimum; TS1, transition state 1; TS2, transition state 2.

The FES was projected against the distance of DCP to the active site (Figure 4A). For simplicity, the global minimum of each FES was adjusted to 0 kcal/mol. In both systems, the global minimum corresponded to some region in the middle of the p1 tunnel (*d1* ≈ 5–6 Å), and not in the active site. From a previous study (Marques et al., 2017), we know that the length of p1 tunnel varies between ≈ 9 and 13 Å (associated with *d1*), which can roughly define the limits of the tunnel mouth (Figure 1). This corresponds well with the local minima at ≈ 12 Å for DhaA31, and ≈ 10 and 13 Å for DhaAwt (Figure 4). Two-dimensional FES profiles can be projected for any set of parameters, which might be useful to assess the potential degeneracy of the CV used (e.g., path CV and *d1*, Figure S14). DhaA31 presented one very steep energy barrier of 4.81 kcal/mol between the global minimum (at 5.94 Å) and a second minimum at the tunnel mouth (12.14 Å), while DhaAwt seems to have a rather smoother and stepwise transport process with two lower energy barriers (2.26 and 1.46 kcal/mol) to reach the tunnel mouth. This fact can have a strong impact on the product release kinetics (*k*_off_), since the transitions between states with lower barriers can occur exponentially faster. This result is consistent with the kinetic studies described above that showed slower DCP unbinding rates with DhaA31 than with DhaAwt.

### Structural Analysis of the Free Energy Landscapes

Here we aimed at understanding structurally the meaning of the different states if the calculated FES and identifying potential hot-spots for improving the unbinding rate of DCP from DhaA31. The FES calculated for the release of DCP through the main tunnel of DhaA31 and DhaAwt have in common quite similar locations of the global minima and their shapes for longer distances. However, they differ significantly in the number of local minima and the heights of the different energy barriers along the pathway. Several relevant stages of the FES have been identified, and the respective simulation frames extracted (Table S3). The respective ensembles can be considered as representative structures of the main states of the systems along the process of DCP unbinding through the main tunnels of DhaA31 and DhaAwt (Figure 4). One first observation reveals that DCP was more confined within DhaA31 than DhaAwt, where it was more especially at the first transition state TS1 (Figure 4). Moreover, the states with *d1* ≥ 13 Å contain DCP outside of the tunnel, where it forms interactions with the residues at the tunnel mouth, before it can be fully released to the bulk solvent (last state, for *d1* ≥ 20 Å). A closer look at the enzymes' structures during the simulations revealed that some of the tunnel-lining residues were highly flexible and presented diverse conformations, which allow the ligand transport through the tunnel. One of such residues is F149, which clearly had two states, observed in both DhaA31 and DhaAwt: (i) the aromatic ring either pointed toward the middle of tunnel, or (ii) it pointed toward the side of the structure under the α4 helix (Figure S15). Because of these two conformations, F149 may play the role of gatekeeper to the transport of ligands through the p1 tunnel.

**Figure 5:**
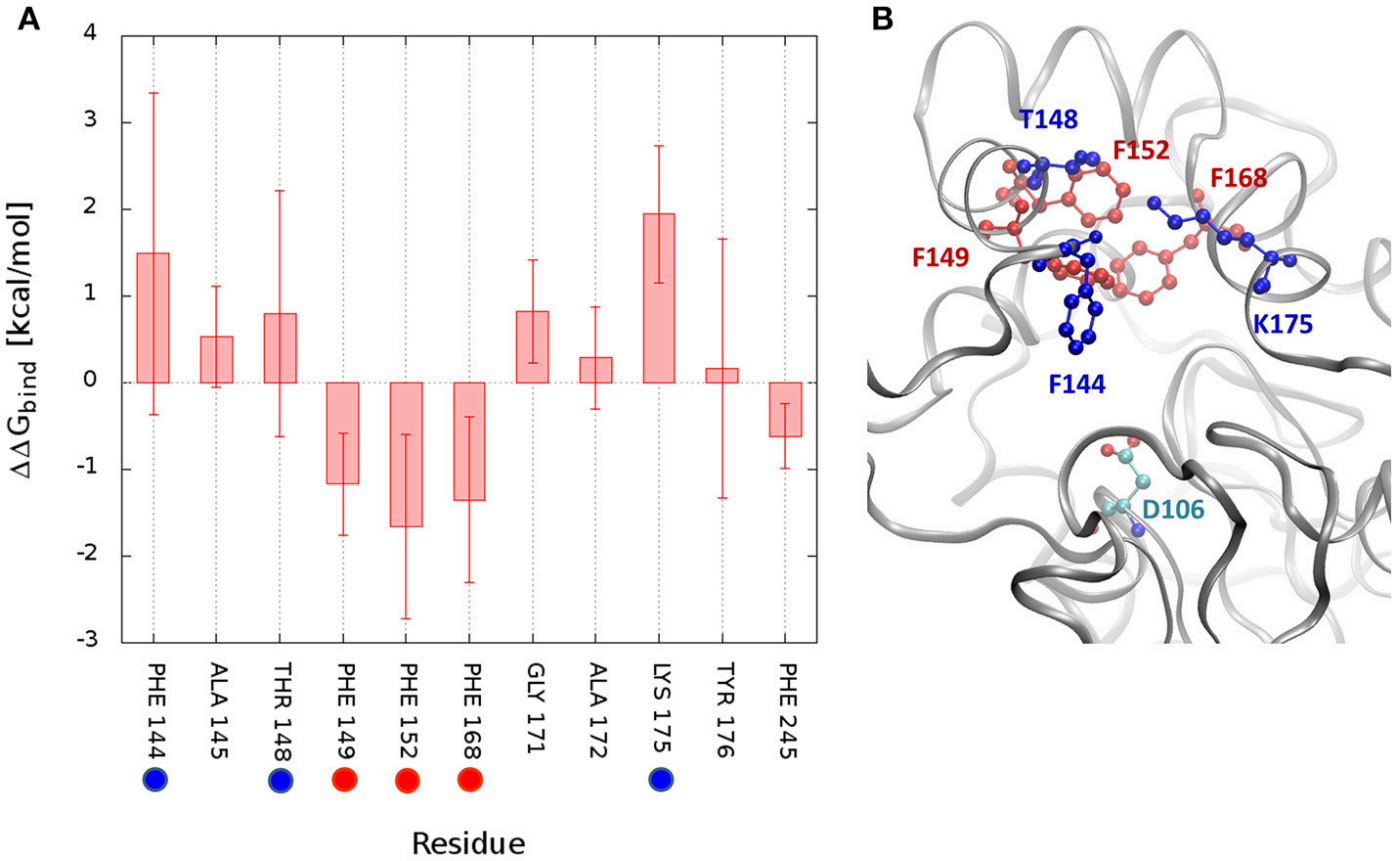
Difference in interaction energies of DCP between the global minimum and the transition state TS1. The binding energy difference (ΔΔG_bind_ = ΔG^min^ – ΔG^TS1^) decomposed by residues for DhaA31 **(A)**, and the structure showing the residues with greatest differences **(B)**. The highlighted residues represent potential hot-spots for decreasing the energy barrier in the unbinding of DCP: (i) by decreasing the interactions of DCP at the global energy minimum with respect to TS1 (red residues), or (ii) by decreasing the energy of TS1 (blue residues). The average interaction differences are represented by the solid bars and the SD by the error bars. Only residues with ΔΔG or SD values ≥ 0.5 kcal/mol are displayed. The catalytic D106 is also represented (green residue).

The measurement of the interactions formed by DCP with each residue may provide a quantitative assessment of what was discussed before and confirm the pivotal role of some residues during the unbinding process. Therefore, the free energy of binding (ΔG_bind_) of DCP was calculated for the structural ensembles. The average interaction energies were calculated for the global minimum energy (Figure S16) and for the TS1 clusters (Figure S17). As expected, at the minimum energy the residues at the tunnel bottleneck dominated the interactions with DCP. The high standard deviations (SD) found for several residues reflect how diverse the structures within the same cluster were. We also tried to assess which residues contributed the most to prevent the transition from the global minima to TS1 due to the strength of their interactions with DCP. For that, we calculated the difference in binding energy between those two states. For DhaA31, the residues with the strongest influence (most negative ΔΔG_bind_) were F152 > F168 > F149 > F245 (Figures 5, S18). These are potential hot-spots for decreasing the energy barrier in DhaA31 and thus improve the unbinding rate of DCP. The residues F144, T148, and K175 form strong interactions in TS1, and might also reveal interesting hot-spots for mutagenesis (Figure 5). The hypothesis here is that the energy barrier may be lowered by increasing the interactions at TS1.

### CaverDock Calculations

Here we wanted to test the ability of a computationally cheap method for predicting the release of DCP from the buried active sites of DhaA31 and DhaAwt, and compare the results with those obtained from the robust methods used above. CaverDock (CaverDock, 2018; Filipovič et al., 2018) was selected for that task. This is a computer program developed for the rapid prediction of the trajectory and energy profile of a ligand being transported through a molecular tunnel. This tool, based on molecular docking, can be used for the fast assessment or high-throughput screening of potential substrates, drugs or metabolites that are expected to bind, or be transported through the tunnels of biomolecules (Pinto et al., 2018; Vávra et al., 2018).

**Figure 6:**
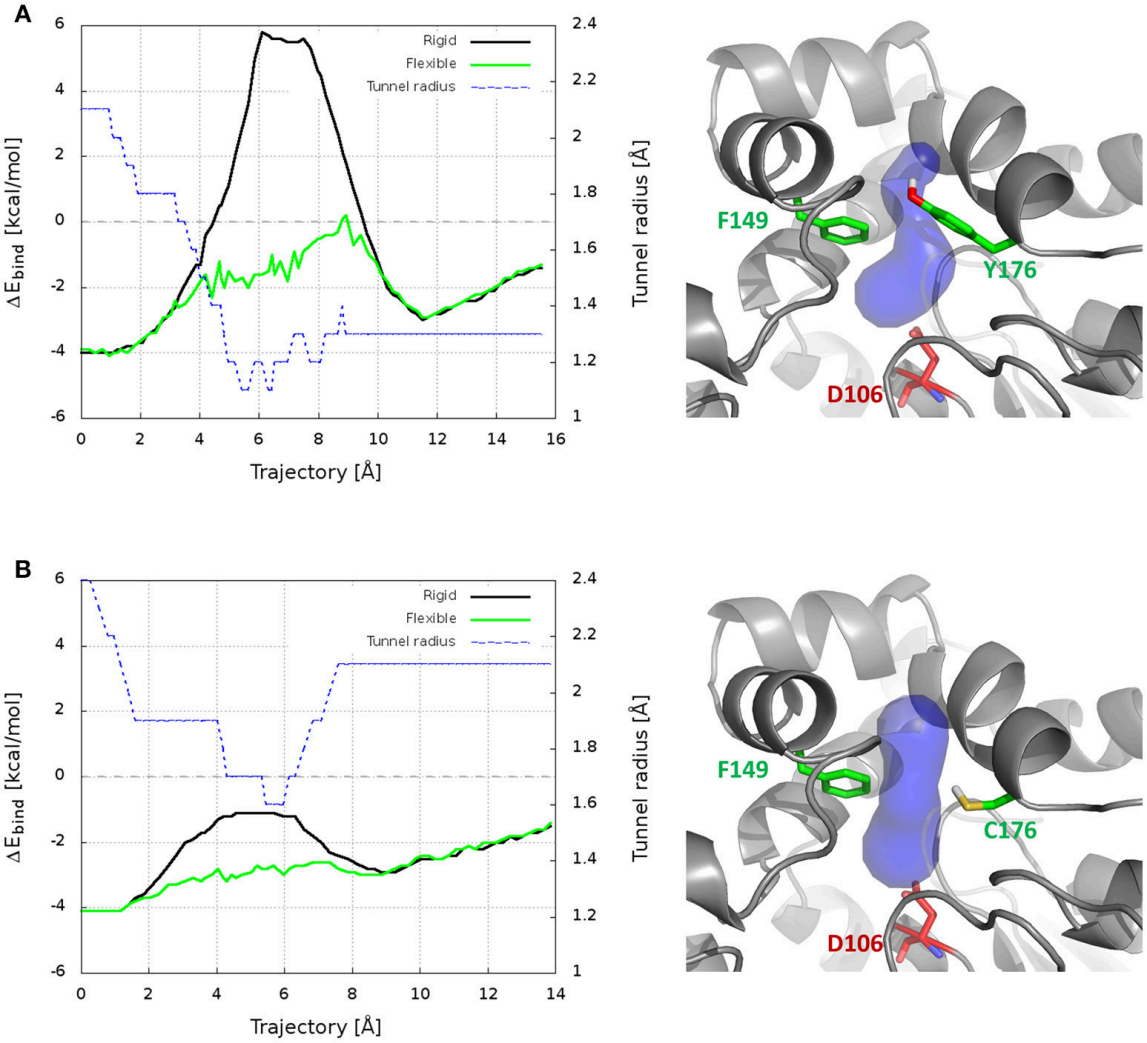
Results from CaverDock calculations on the transport of DCP through the main tunnel of DhaA31 **(A)** and DhaAwt **(B)**. On the left are shown the binding energy (ΔE_bind_) profiles for the rigid and flexible receptor calculations, and the tunnel radius; the trajectory is directed from the active site to the surface. On the right are the respective enzyme structures (gray cartoon), with the p1 tunnel (blue surface), the residues made flexible (green sticks) and the catalytic nucleophile (red sticks).

CaverDock was used here to predict the trajectories and energy profiles of DCP through p1 tunnel of DhaA31 and DhaAwt, which were compared with the ones obtained from the robust MTD method. The results showed that the two enzymes have energy minima with similar binding energy (ΔE_bind_ = −4.1 kcal/mol), located at the active site instead of the middle of the tunnels (Figure 6 and Table S4), which is in contrast with the FES obtained from the funnel-MTD. When the calculations were performed with static receptors, DCP showed very high and repulsive energy barrier (with ΔE_bind_ = +9.9 kcal/mol) for DhaA31, which was in great contrast with DhaAwt that had lower barrier (3.1 kcal/mol) and always favorable energies (ΔE_bind_ < 0). This was due to the clashes of DCP with the protein residues passing through the narrower tunnel of DhaA31. The energy barrier was much higher for DhaA31 than for DhaAwt, which is qualitatively in agreement with the FES profiles (Table S4). When the CaverDock calculations were performed with flexibility (a feature still under development), the energy profiles became smoother and the energy barriers for the unbinding of DCP dropped significantly, to 4.3 kcal/mol for DhaA31, and 2.7 kcal/mol for DhaAwt. The residues that are made flexible are selected by the extent of clashes during the rigid docking. In this case, they were F149 and Y176 for DhaA31, and F149, and C176 for DhaAwt. These residues are located at the tunnel bottleneck, and they were shown to form strong interactions with DCP at the global energy minima identified in the FES, thus confirming their importance (Figure S16). This result is remarkable, especially when considering the dramatic difference in the calculation costs for CaverDock (hours) and the free energy methods (weeks).

## Discussion

The calculation of the kinetics and thermodynamics of ligand (un)binding has recently shown to be pivotal in drug design, but it can also be important in structural biology and biocatalysis. This is the case of the mutant enzyme DhaA31, which is currently the best known HLD for hydrolyzing the genotoxic compound TCP, but whose catalytic turnover number is limited by the release of the DCP product. For this system, the assessment of the kinetic and thermodynamics bottlenecks in the unbinding of DCP may pave the way to the design of improved biocatalysts.

In this study we have calculated the unbinding kinetic rates (*k*_off_) of DCP from the active sites of two enzymes, DhaA31, and DhaAwt, using two different methods—metadynamics (MTD) and adaptive sampling. Both methods predicted faster unbinding rates from DhaAwt than from DhaA31 (Table 1), which is in good agreement with previous evidence (Marques et al., 2017). However, there were considerable differences in the results from those two methods. For each system, the *k*_off_ values differed by 1-2 orders of magnitude, being slower with the MTD method than with the adaptive sampling. Regarding the relative values of the *k*_off_ rates, we obtained DhaAwt/DhaA31 ratio of 36 with MTD and only 2.5 with adaptive sampling, meaning that the latter method predicted faster rates for the two systems, but also closer values for those enzymes. On the other hand, the precision of the *k*_off_ values obtained with adaptive sampling was higher (the relative errors were lower) than with the MTD method. Previous studies have attributed the differences between the predicted and experimental *k*_off_ values to the errors in the force fields, the lack of polarizability, or the existence of tautomers, among other factors (Tiwary et al., 2015; Ferruz and De Fabritiis, 2016; Bruce et al., 2018). The simulations were performed under the same force field and water model (ff12SB and TIP3P, respectively), and therefore these differences are probably due to the intrinsic differences in the two methods. Since both methods have different types of biases—the MTD relies on a bias of repulsive potential energy deposited based on the CV defined by the user, while the adaptive sampling uses Markov state models, calculated on-the-fly upon a user-defined metrics to start new epochs of MDs—and there are no experimental values available, it is difficult to assess which results are more accurate. Moreover, it is known that MDs performed with the same force fields but using different software packages may produce different conformational ensembles, and consequently different results (Childers and Daggett, 2018). The effects of the force field and solvent on the predicted kinetic rates were tested by additional adaptive sampling simulations performed with the ff14SB force field and OPC3 water model (Table 1). When compared to the available experimental data, the *K*_d_ value predicted for DhaAwt using the combination ff14SB+OPC3 (8.3 ± 2.5 mM) was the closest to the experimental value (*K*_d_ = 0.95 ± 0.34 mM). This seems to suggest that these conditions can better represent the physical properties of that system. A deeper discussion on this topic is presented in Discussion S2 in Supplementary Material. We have demonstrated that the choice of the method, force field and water model can have a high impact on the prediction of kinetic properties. However, important conclusions could consistently be inferred from the comparative study of the two systems, namely the higher propensity of DhaAwt to release DCP as compared to DhaA31. This strongly supports the value of comparative studies with similar systems, namely for the design of new enzyme variants in protein engineering.

The funnel-MTD simulations provided the free energy profiles for the unbinding of DCP from DhaA31 and DhaAwt, which allowed us to derive some conclusions about the respective energetic barriers and bottlenecks. The global energy minima in both enzymes were found in the middle of the tunnel (for *d1* ≈ 5-6 Å). After the global minimum, DhaA31 presented one steep energy barrier of 4.81 kcal/mol before DCP could reach the tunnel mouth, while DhaAwt had two steps with considerably reduced barriers (with 2.26 and 1.46 kcal/mol, respectively). This implies faster unbinding kinetic rates for DhaAwt, which is in good agreement with the kinetic calculations. The first transition state intermediate (TS1) in DhaA31 also corresponds to the geometric bottleneck and presents much higher steric constraints than the one observed in DhaAwt (Figure 4). The structural clusters, corresponding to the significant state along the FES, allowed the assessment of the respective binding energy of DCP with the protein's residues. From this analysis it was possible to identify the residues that interact with DCP at the energy minima and transition states, and thus contribute to the stabilization of these states. We hypothesize that the difference in binding energy between the global energy minimum and the transition states may help identify the residues that contribute the most to retain DCP in that minimum and prevent the enzyme-product complex to proceed further to the full unbinding. Therefore, the residues with more negative ΔΔG_bind_ are the most likely hot-spots for improving the unbinding rates. In DhaA31, there residues correspond to F152, F168, F149, and F245 (Figure 5). It is known, however, that residues F149 and F245 are important to stabilize and orient TCP toward the S_N_2 step (Marques et al., 2017), and therefore they should not be mutated to avoid undesirable disruption of the chemical steps. Previous studies performed with this system have identified several of these bottleneck residues as highly interacting with DCP, e.g., F149, F152, and F168 (Marques et al., 2017). However, their role was not so clear, and the current analysis came to confirm their pivotal importance in preventing the transition from the energy minimum toward the release. The residues with strongest interactions at the transition state, F144, T148, and K175, also represent interesting hot-spots to decrease the energy of TS1 and promote the transition of DCP along the unbinding process. However, the results from this approach are more unpredictable since the entropy can also be highly affected. CaverDock provided very interesting insights into the transport of DCP through the tunnels, especially considering that it is fast and has very low hardware requirements. With these simple calculations it could be concluded that DhaA31 has higher energy barrier to the unbinding of DCP as compared with DhaAwt, and we identified some of the residues that may hinder the transport the most.

Overall, we have shown that the computation of the kinetics and thermodynamics of protein-ligand unbinding can be a powerful tool for protein engineering when the goal is to improve the unbinding rates of a ligand from a biomolecule. Similar methods used can also be applied when the aim is to improve the ligand (substrate) binding. We illustrated that even highly sophisticated methods cannot precisely estimate kinetic values due to the computational limitation, and the results may highly depend on the selected parameters. However, they can be very useful for comparative purposes, which are typically needed in protein engineering projects. The free energy computation with funnel-MTD, or other enhanced-sampling free energy methods, can provide a deep insight into the binding/unbinding process, allow identification of the critical stages energetic and disclose the key residues for the unbinding. On the other hand, CaverDock is very fast and user-friendly, yet it may provide significant information about the ligand transport and enable the identification of key residues to improve the ligand transport. Different strategies can be followed for engineering new enzymes with improved ligand unbinding kinetic rates. The potential hot-spots for mutagenesis can be selected based on: (i) the residues showing the highest interaction differences between the energy minimum and transition state—aiming to decrease the energy barrier; (ii) residues interacting at the transition state—aiming to decrease the transition state energy; (iii) tunnel-lining residues—aiming to change the shape and geometric bottleneck of the tunnel; and (iv) residues in contact with the tunnel-lining residues—aiming to change the flexibility and dynamic properties of the tunnel residues. The selected hot-spot residues can be targeted by site-directed mutagenesis, smart libraries or saturation mutagenesis. The effects of particular mutations on the unbinding rates can be anticipated with *in silico* calculations, either with the thorough but costly approaches (MTD or adaptive sampling), or using the cheaper CaverDock for a faster screening.

## Conclusions

Here we reported the application of metadynamics and adaptive sampling for computationally estimating the unbinding rates of the DCP product from two enzymes, DhaA31 and DhaAwt, and for aiding the design of improved biocatalysts. The unbinding of DCP is the rate-limiting step in the catalytic conversion of the toxic TCP with DhaA31, and improving this rate has biotechnological importance. Free energy calculation confirmed the different energetic profiles in the release of DCP by the two enzymes, and provided insights into the energetic bottlenecks in the unbinding process. By analyzing the interactions of DCP with DhaA31 at the critical stages we have identified several hot-spot residues that can be targeted by mutagenesis. Strikingly, some of these hot-spots were identified by the far less demanding CaverDock tool based on molecular docking. Site-directed mutagenesis or directed evolution applied on those hot-spots may result in new enzyme variants with the ability to release the DCP product at faster rates and thus present enhanced catalytic properties.

## Author Contributions

SM carried out the computational work and wrote the manuscript. All authors contributed to the design of the study, interpretation of the data, and have given approval of the final version of the manuscript.

### Conflict of Interest Statement

The authors declare that the research was conducted in the absence of any commercial or financial relationships that could be construed as a potential conflict of interest.

## Abbreviations

TCP, 1, 2: 3-trichloropropane
DCP, 2: 3-dichloropropan-1-ol
HLD: haloalkane dehalogenase
MTD: metadynamics
funnel-MTD: funnel-metadynamics
CV: collective variable
RMSD: root-mean-square deviation
MD: molecular dynamics
HTMD: high throughput molecular dynamics
FES: free energy surface
τ: average transition time
MSM: Markov state model
SD: standard deviation.
