# Supplementary material for: Computational Study of Protein-Ligand Unbinding for Enzyme Engineering
Source: Front Chem. 2019 Jan 8;6:650. doi: 10.3389/fchem.2018.00650 (PMC6331733; doi:10.3389/fchem.2018.00650)
Supplement: Supplementary file 1 [file Table_1.docx]

Supplementary Material

**Computational study of protein-ligand unbinding for
enzyme engineering**

Sérgio M. Marques^1,2^, David Bednar^1,2^, Jiri Damborsky^1,2^

^1^Loschmidt Laboratories, Department of Experimental Biology and Research Centre for Toxic Compounds in the Environment RECETOX, Faculty of Science, Masaryk University, Kamenice 5/A13, 625 00 Brno, Czech Republic

^2^International Clinical Research Center, St. Anne's University Hospital Brno, Pekarska 53, 656 91 Brno, Czech Republic

***Table of content***

*Supplementary Figures ……………………………………………………….……….….. p. 3*

Supplementary Figure S1. Construction of the path collective variable for the release of DCP.

Supplementary Figure S2. Unbiased release times (*t*) of DCP.

Supplementary Figure S3. Empirical cumulative distribution function and probability density for the full unbinding metadynamics simulations of DCP.

Supplementary Figure S4. Transition dissociation time estimated with increasing number of simulation runs.

Supplementary Figure S5. Chapman-Kolmogorov test result for the Markov state models from simulations using ff12SB force field and TIP3P waters.

Supplementary Figure S6. Chapman-Kolmogorov test result for the Markov state models from simulations using ff14SB force field and TIP3P waters.

Supplementary Figure S7. Chapman-Kolmogorov test result for the Markov state models from simulations using ff14SB force field and OPC3 waters.

**Supplementary Figure S8.** Combined RMSD plots for the adaptive sampling simulations.

Supplementary Figure S9. Visualization of the funnel restraints.

Supplementary Figure S10. Variation of the distance of DCP to the active site during the funnel-MTD simulations.

**Supplementary Figure S11.** RMSD plots for the funnel-MTD simulations.

Supplementary Figure S12. Evolution of the energy difference between the first two basins.

Supplementary Figure S13. Evolution of the energy barrier between the first two energy minima.

Supplementary Figure S14. Two-dimensional free energy surfaces for the release of DCP based on the original CV and the distance of DCP to the active site.

Supplementary Figure S15. Conformations of the F149 residue in closed and open states.

Supplementary Figure S16. Binding free energy of the protein residues with DCP at the global minimum.

Supplementary Figure S17. Binding free energy of the protein residues with DCP at the TS1.

Supplementary Figure S18. Binding free energy difference between the global minimum and the TS1 states.

*Supplementary Tables ……………………………………………………………………. p.17*

Supplementary Table S1. Residues lining the p1 tunnel selected for defining the path collective variables in DhaA31 and DhaAwt.

Supplementary Table S2. Kinetic parameters and statistics obtained for the release of DCP form DhaA31 and DhaAwt.

Supplementary Table S3. Relevant stages of the free energy surface for the unbinding of DCP from DhaA31 and DhaAwt.

Supplementary Table S4. Energy parameters from CaverDock calculations for the transport of DCP through the p1 tunnel.

*Supplementary Discussions ………………………………………….………….……….. p.20*

Supplementary Discussion S1. Setting up the CV used in the MTD calculations.

Supplementary Discussion S2. The effects of the force field and solvent model in the calculation of kinetic rates.

*References ………………………………………….……………………………….…….. p.21*

***Supplementary Figures***

**A B**


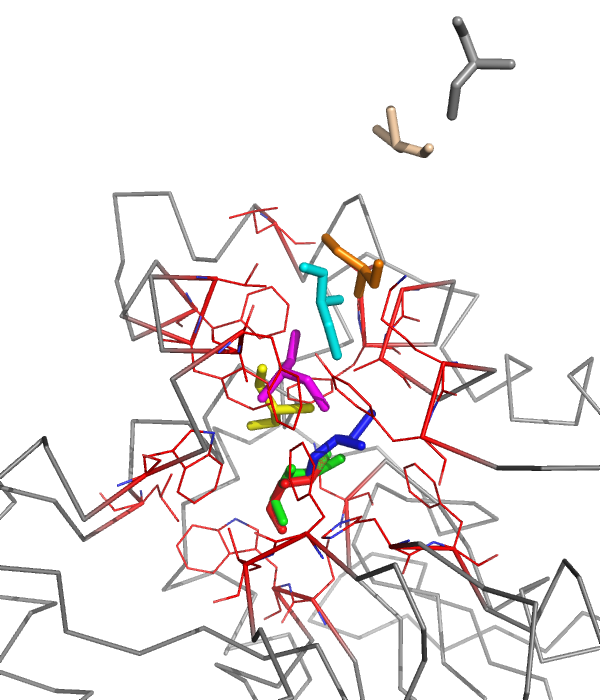

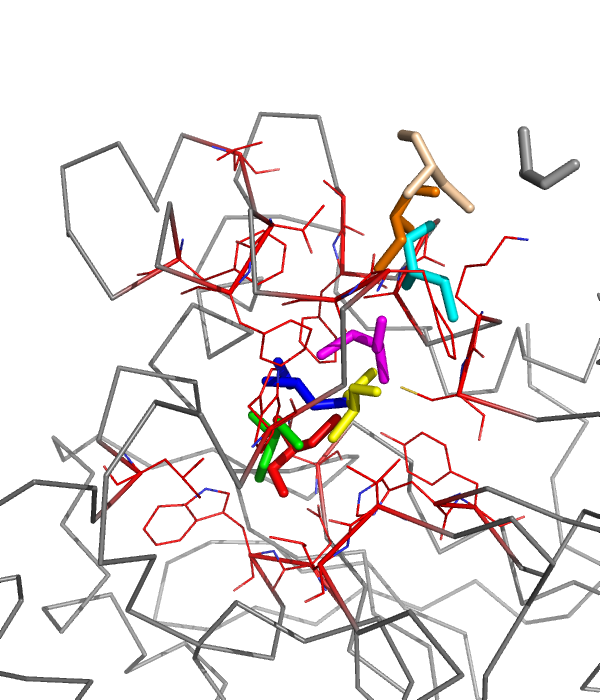


Supplementary Figure S1. Construction of the path collective variable for the release of DCP, for (A) DhaA31 and (B) DhaAwt. The nine frames used for defining of the path variable (*p3*) are composed by DCP (in stick representation, all frames shown) and the residues surrounding the p1 exit pathway (red lines, only first frame shown). DCP with respective *p3* values: red (1), green (2), blue (3), yellow (4), magenta (5), cyan (6), orange (7), wheat (8), grey (9). The proteins backbones are shown for reference as the grey ribbons, and the H-atoms are omitted for clarity.

Supplementary Figure S2. Unbiased release times (*t*) of DCP from the buried active sites of (A) DhaA31 and (B) DhaAwt, obtained from the infrequent metadynamics simulations.

**A**

**B**


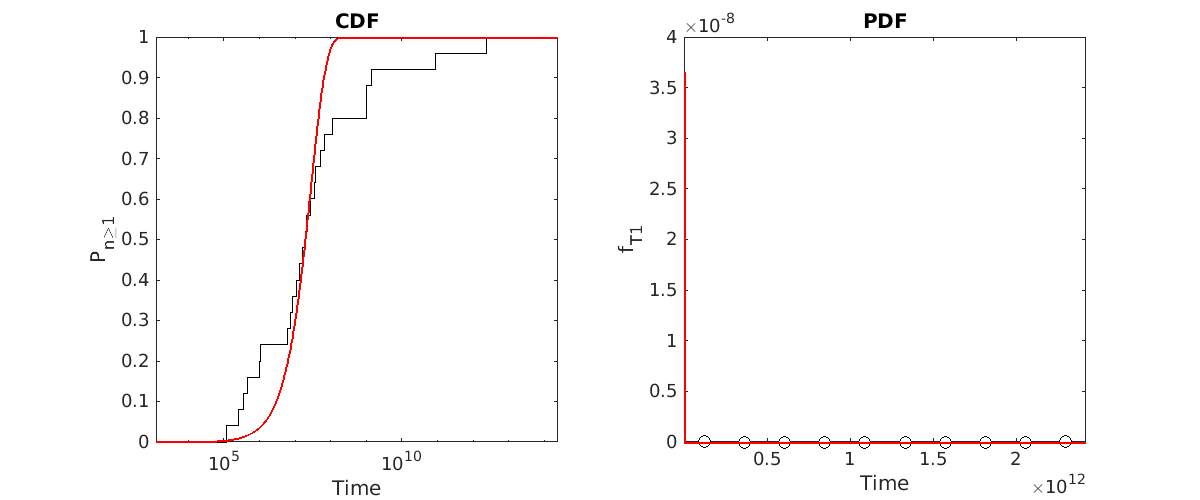


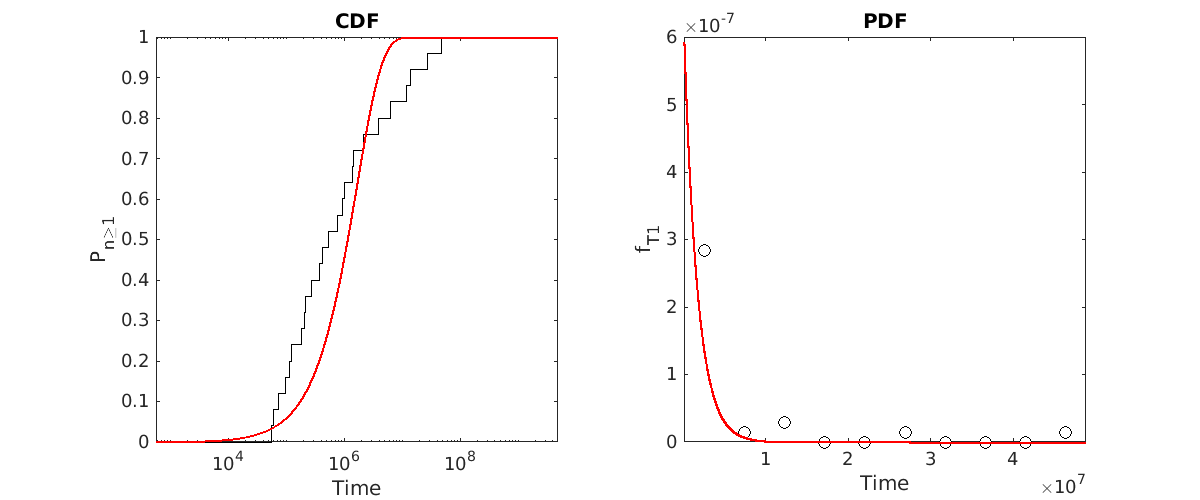


Supplementary Figure S3. Empirical cumulative distribution function (CTF) and probability density (PDF) for the full unbinding metadynamics simulations of DCP from (A) DhaA31 and (B) DhaAwt.

Supplementary Figure S4. Transition dissociation time estimated with increasing number of simulation runs.


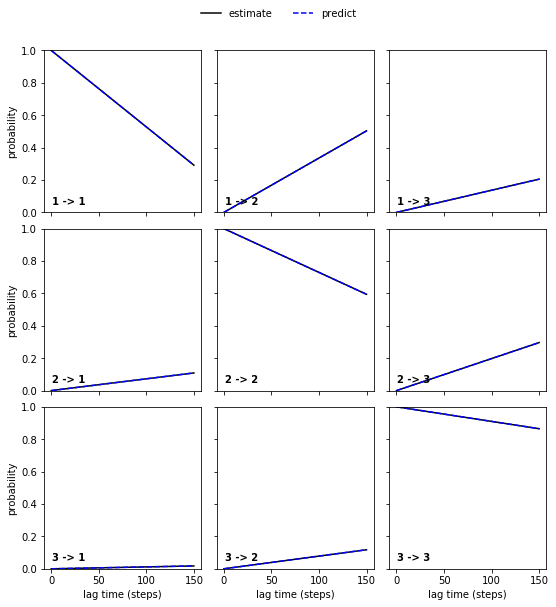

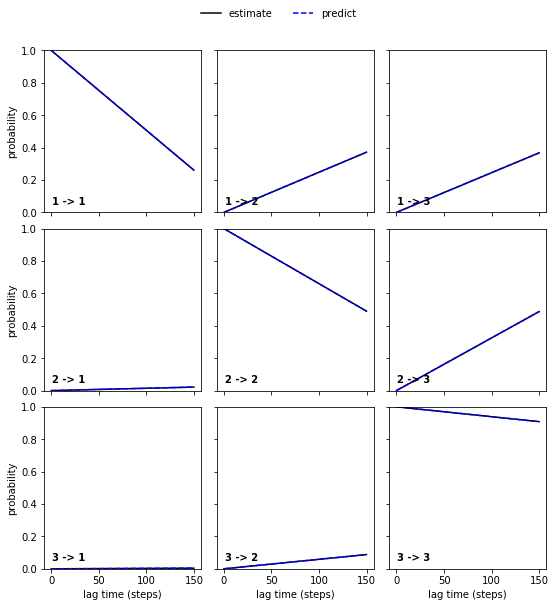


**A B**

Supplementary Figure S5. Chapman-Kolmogorov test result for the Markov state models from simulations using ff12SB force field and TIP3P waters, for A) DhaA31 and B) DhaAwt.

**A B**


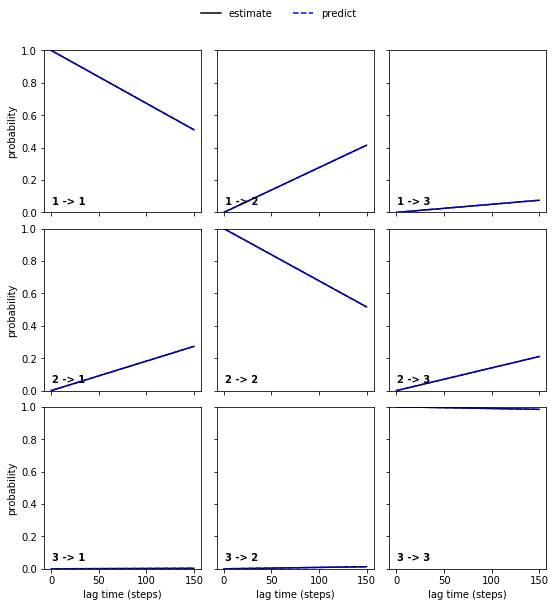

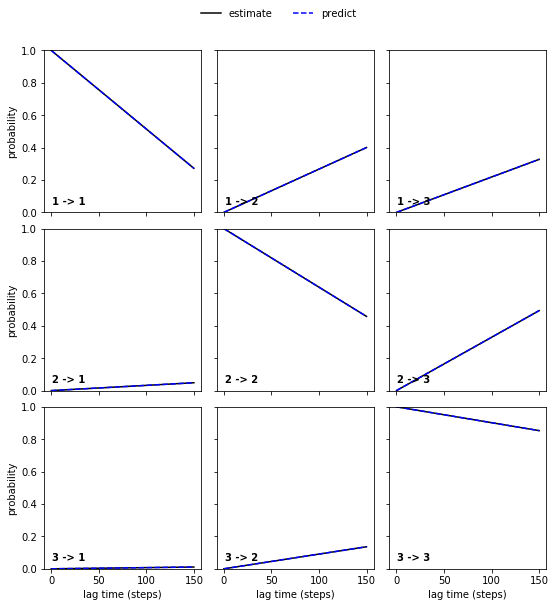


Supplementary Figure S6. Chapman-Kolmogorov test result for the Markov state models from simulations using ff14SB force field and TIP3P waters, for A) DhaA31 and B) DhaAwt.


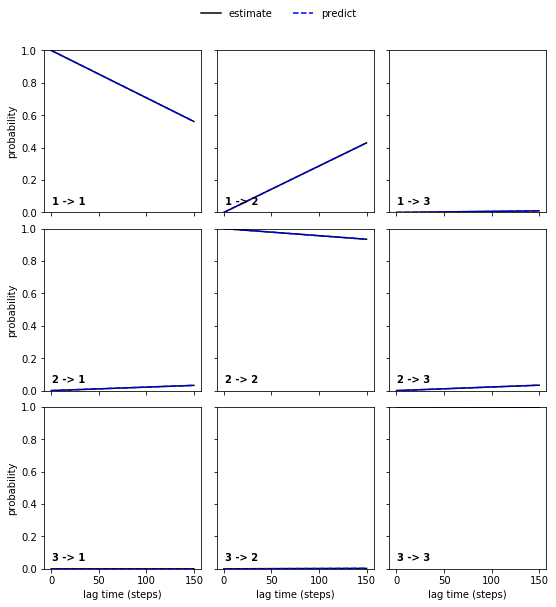

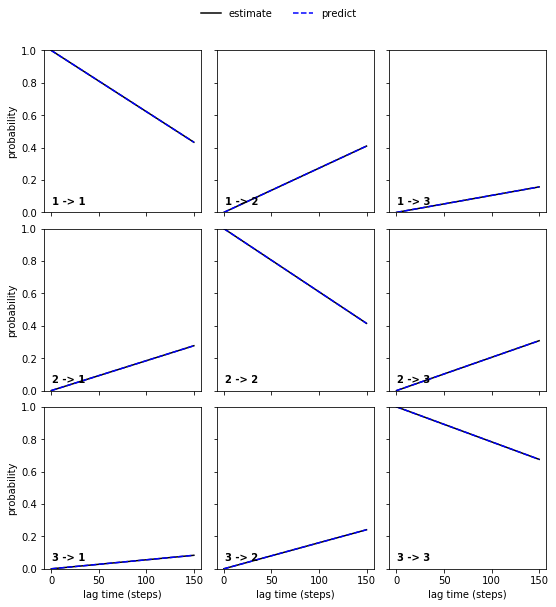


**A B**

Supplementary Figure S7. Chapman-Kolmogorov test result for the Markov state models from simulations using ff14SB force field and OPC3 waters, for A) DhaA31 and B) DhaAwt.

**A**

**B**


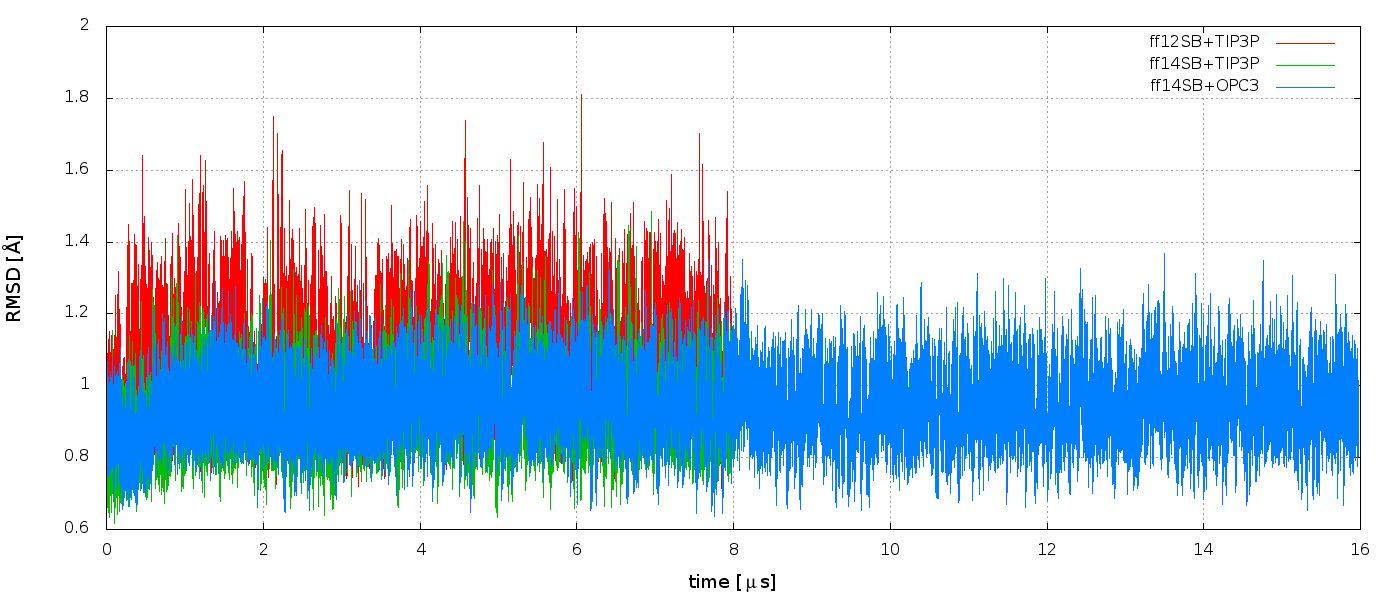


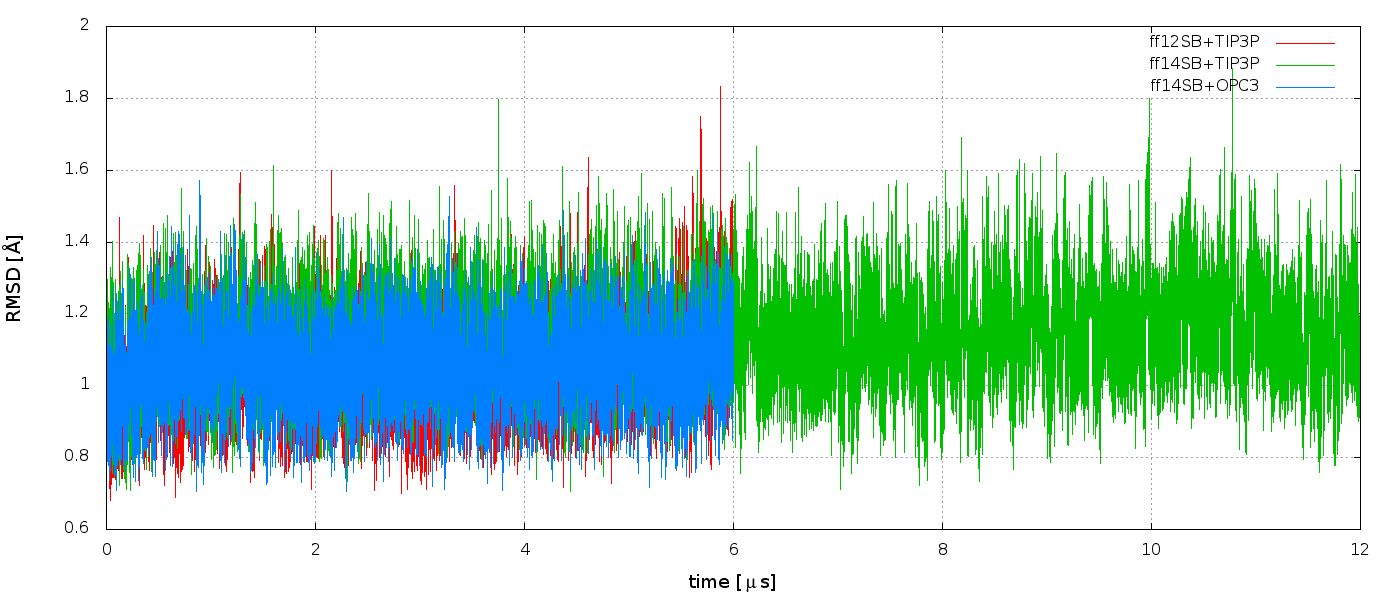


**Supplementary Figure S8.** Combined RMSD plots for the adaptive sampling simulations with DhaA31 (A) and DhaAwt (B), performed with the different force field and water model combinations. The RMSD values were calculated for the backbone atoms of the protein in comparison with the respective crystal structures (to exclude the very flexible terminal residues, only residues 8–288 were used in the RMSD calculation).


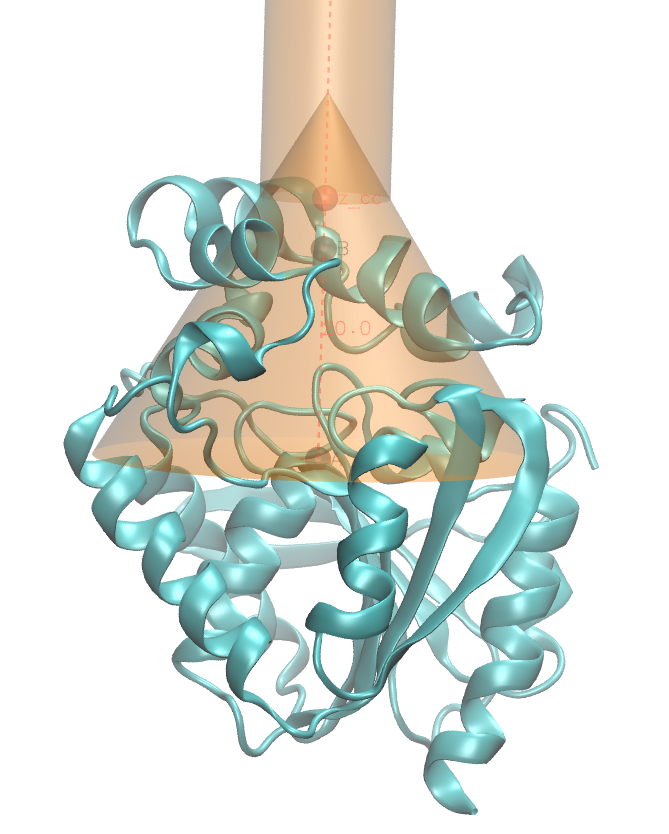

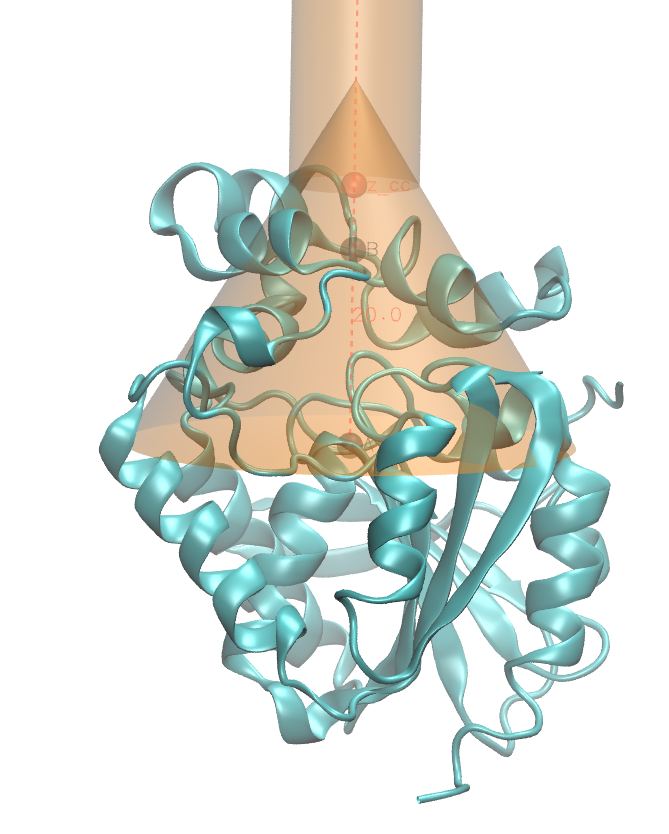


**A B**

**A**

**B**

***Z_cc_***

***Z_cc_***

**B**

**A**

**Supplementary Figure S9.** Visualization of the funnel restraints used with DhaA31 (A) and DhaAwt (B). The funnel axis (red dashed line) is defined by points A and B (black spheres); Z_cc_ (20 Å, is the distance between point A and the red sphere) defines the intersection between the cone and the cylinder; *R_cyl_* (5.0 Å) is the cylinder radius.

**A B**


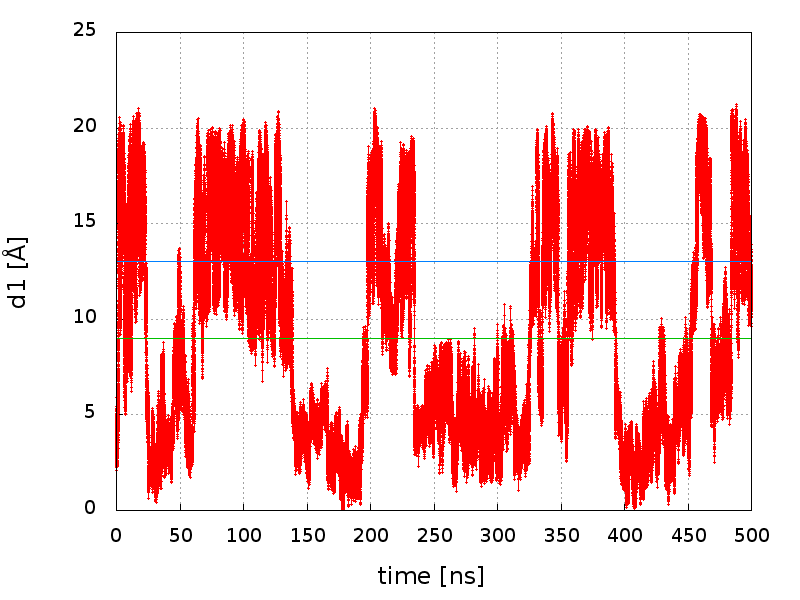

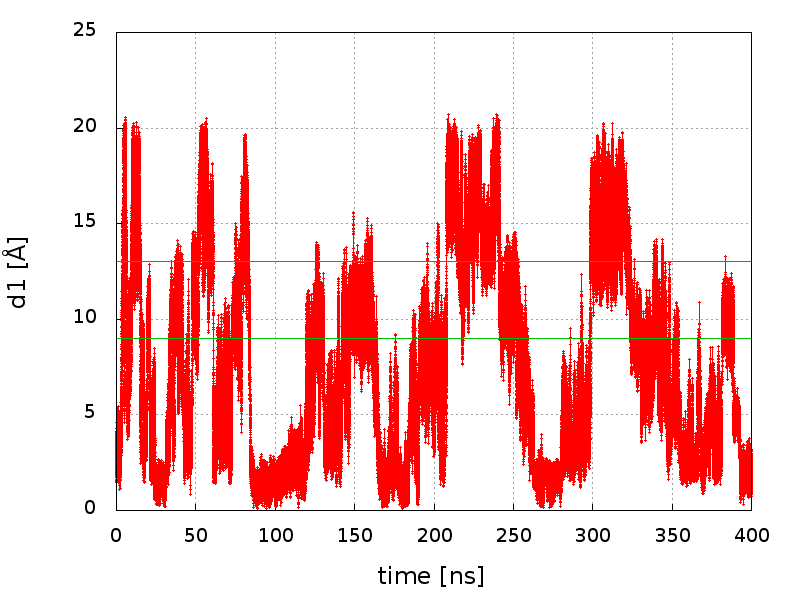


Supplementary Figure S10. Variation of the distance of DCP to the active site during the funnel-MTD simulations with DhaA31 (A) and DhaAwt (B). The horizontal lines represent the lower- (green, 9 Å) and upper-bound (blue, 13 Å) thresholds for the tunnel mouth, for roughly estimating the bound and unbound states.


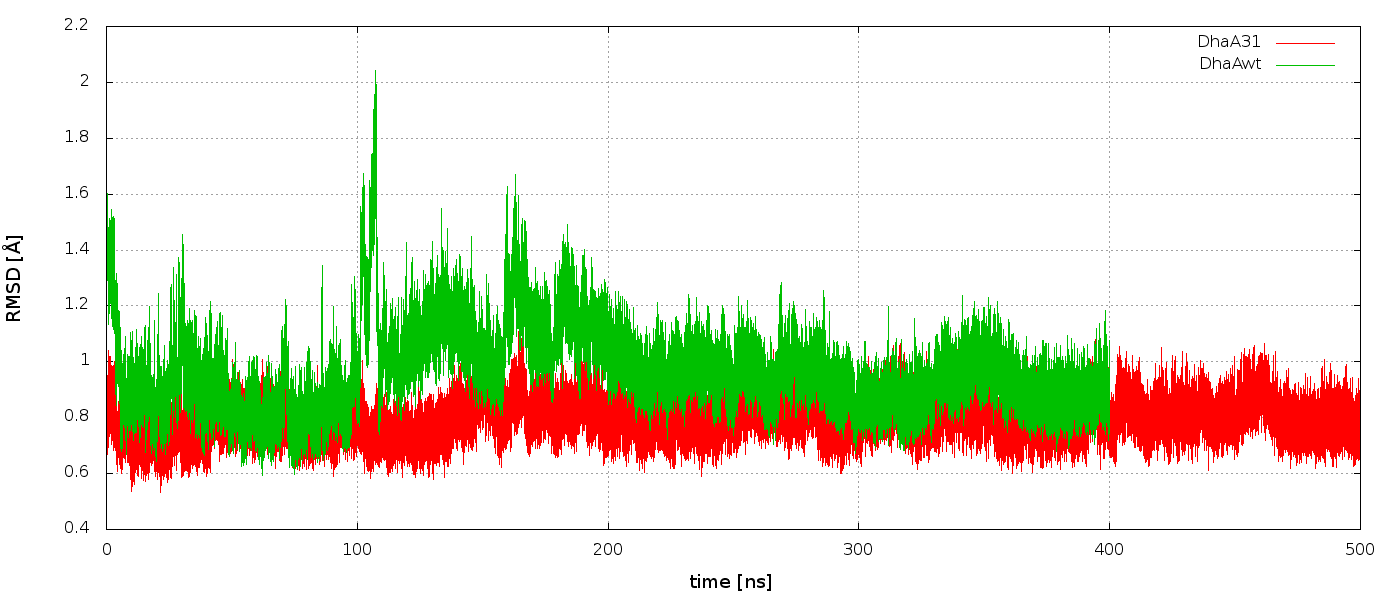


**Supplementary Figure S11.** RMSD plots for the funnel-MTD simulations with DhaA31 (A) and DhaAwt (B). The RMSD values were calculated for the backbone atoms of the protein in comparison with the respective crystal structures (to exclude the very flexible terminal residues, only residues 8–288 were used in the RMSD calculation).

**A B**


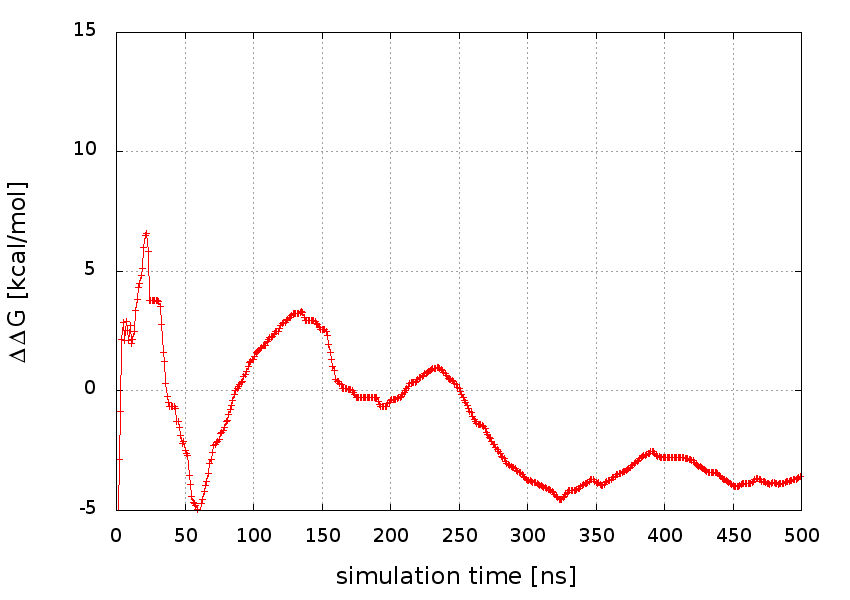

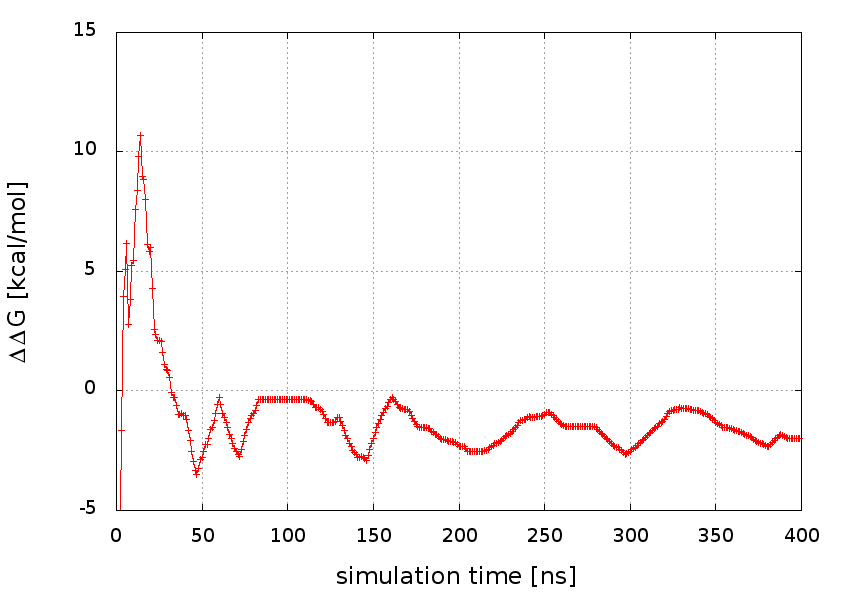


Supplementary Figure S12. Evolution of the energy difference between the first two basins, for DhaA31 (A) and DhaAwt (B). The basins were calculated between the original path CV values *p3.sss*=[3:4.5] and *p3.sss*=[5:7] for DhaA31, and *p3.sss*=[3:5] and *p3.sss*=[5.5:7] for DhaAwt.


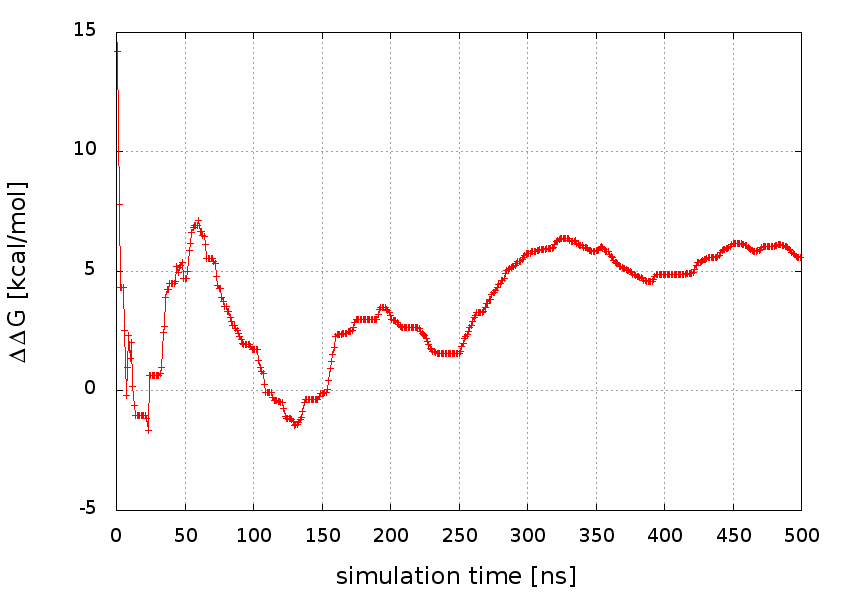

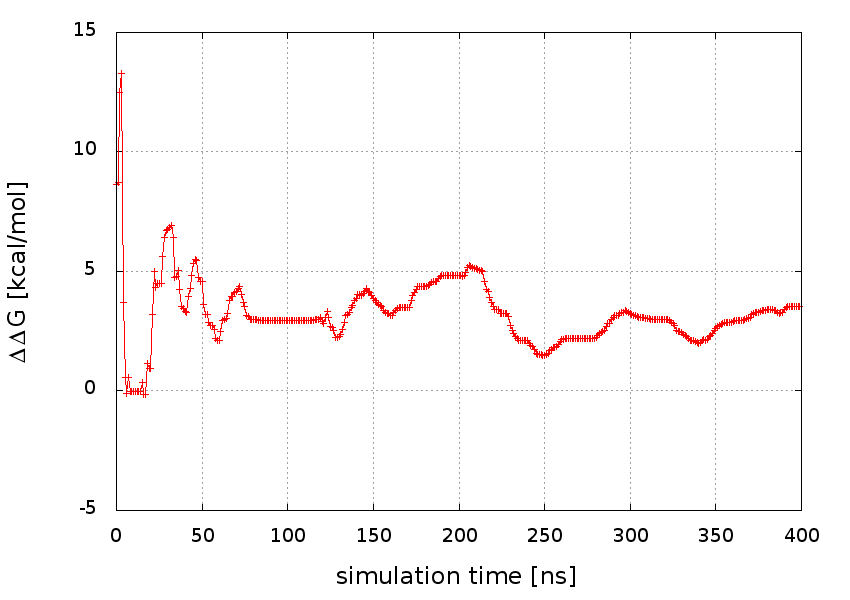


**A B**

Supplementary Figure S13. Evolution of the energy barrier between the first two energy minima, respectively at *d1* ≈ 6 and 10 Å, for DhaA31 (A) and DhaAwt (B).

**A B**


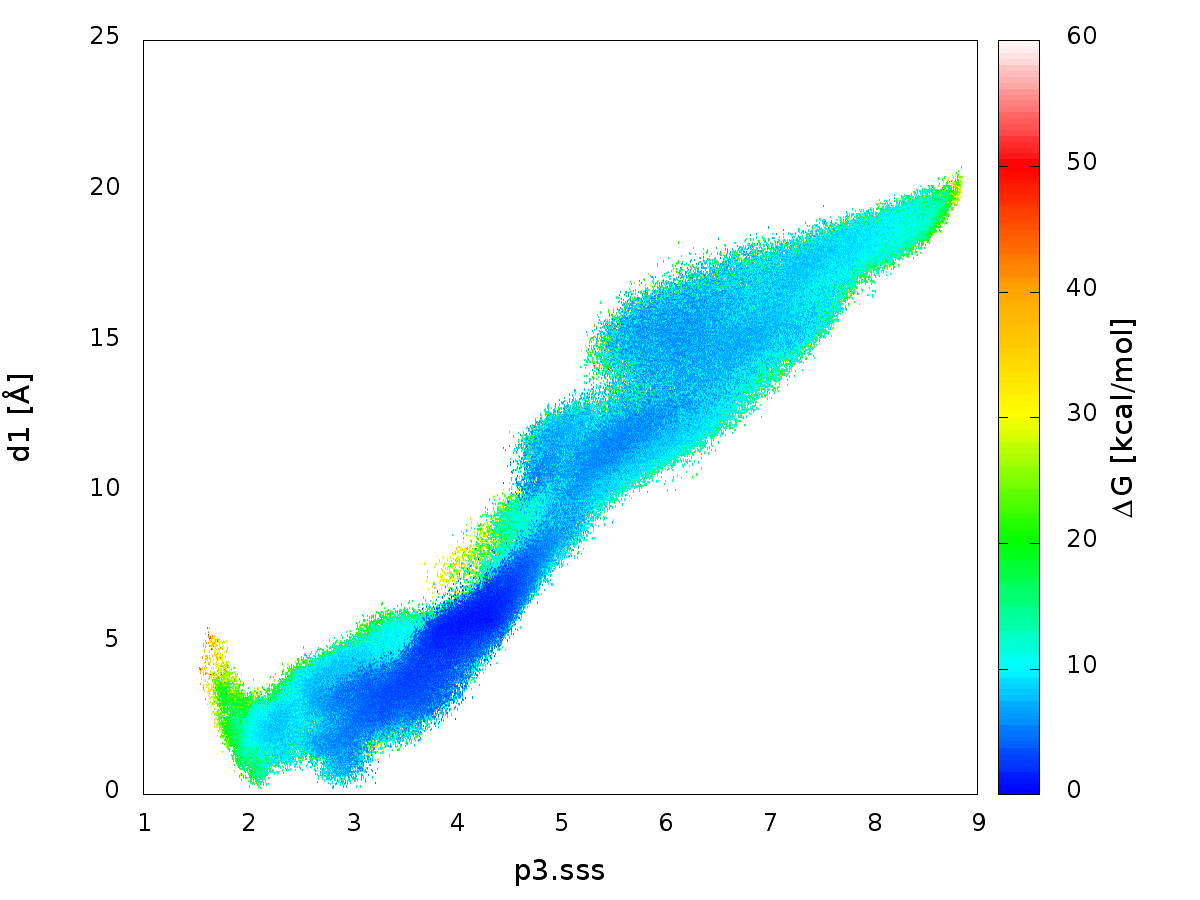

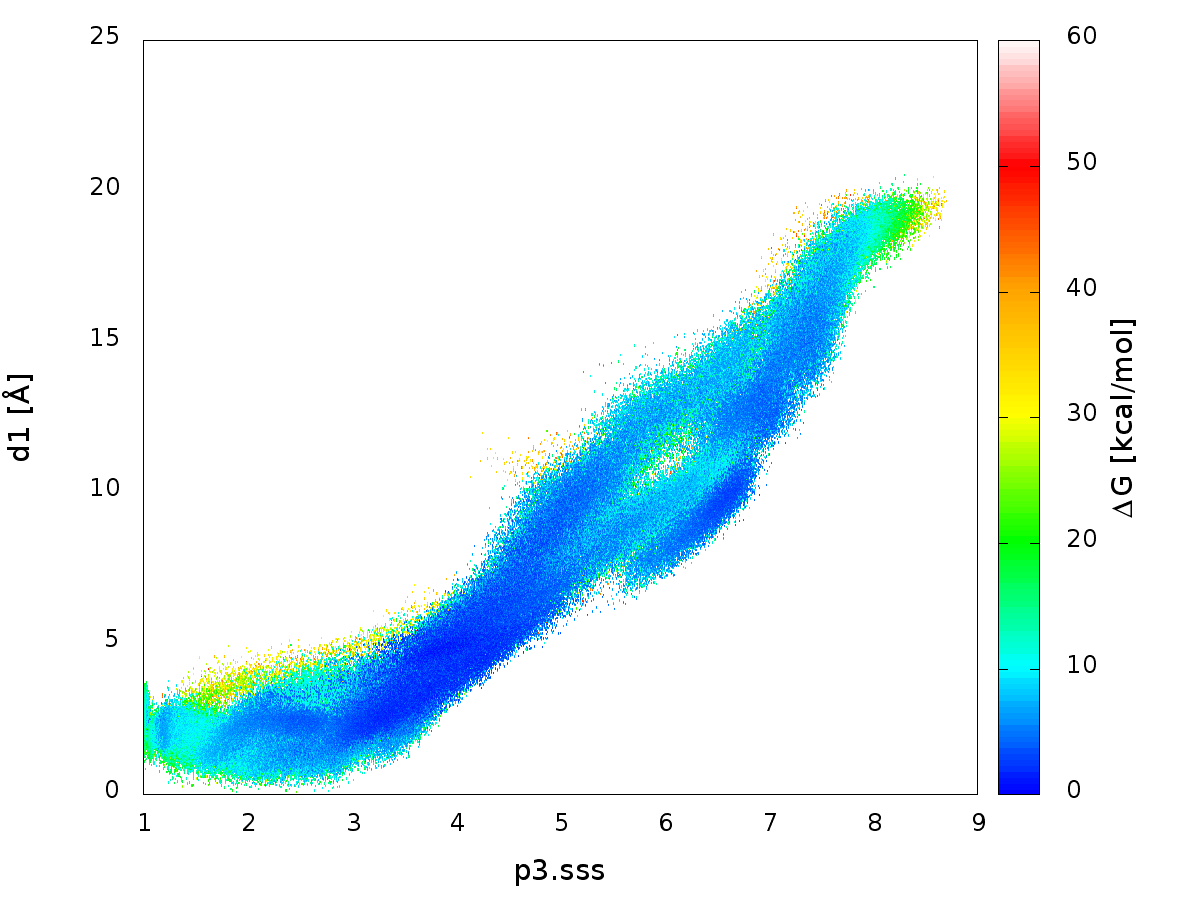


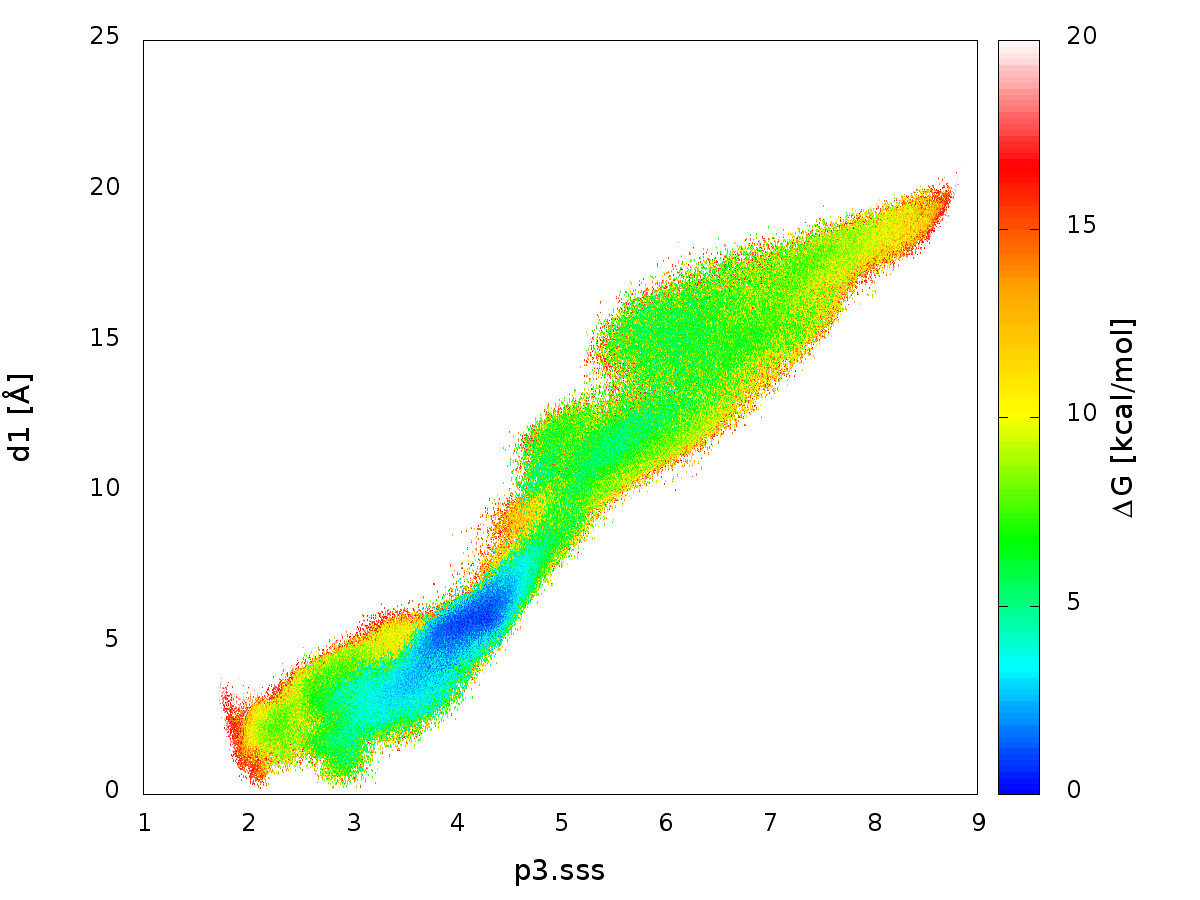

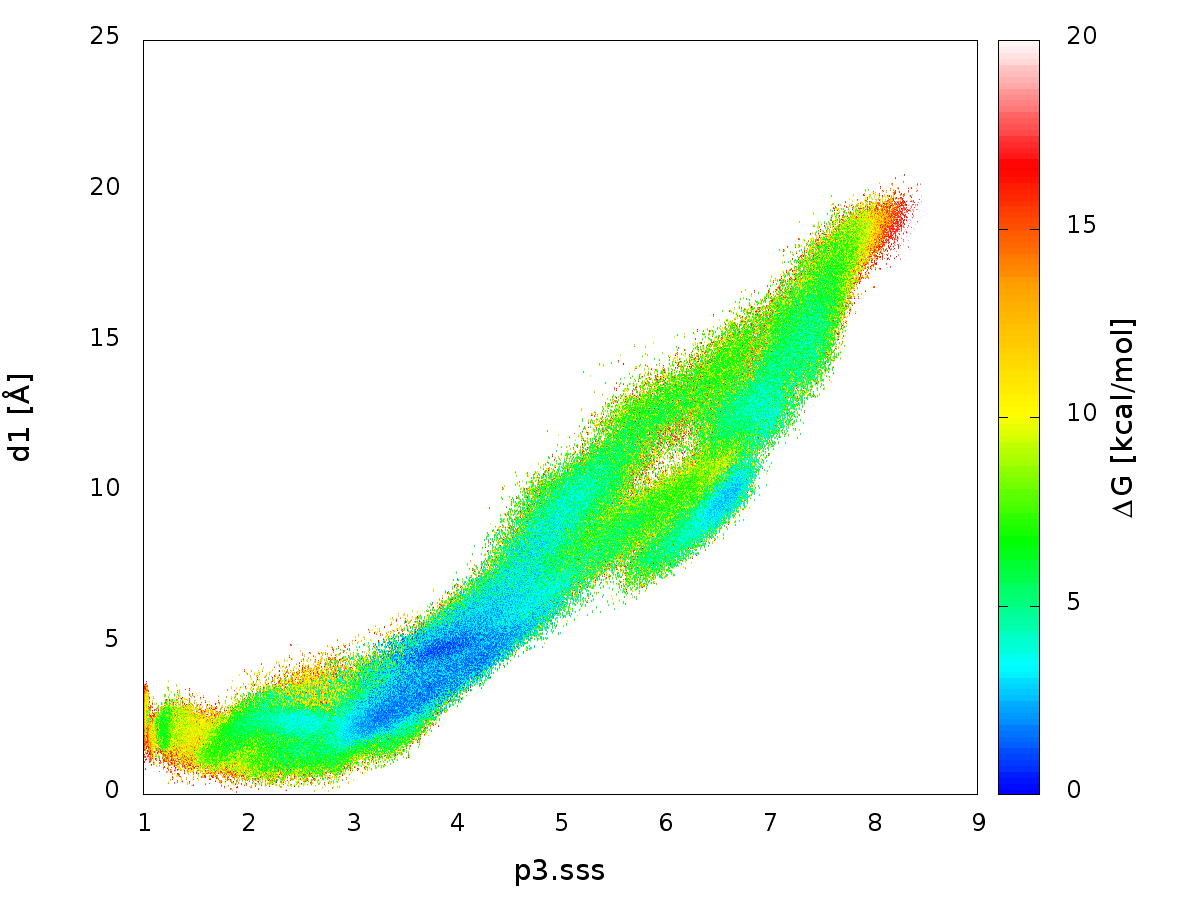


Supplementary Figure S14. Two-dimensional free energy surfaces (FES) for the release of DCP based on the original CV *(p3.sss*) and the distance of DCP to the active site (*d1*), for DhaA31 (A) and DhaAwt (B). The top row Supplementary Figures show the global FES and the bottom ones show more detailed FES for ΔG ≤ 20 kcal/mol.


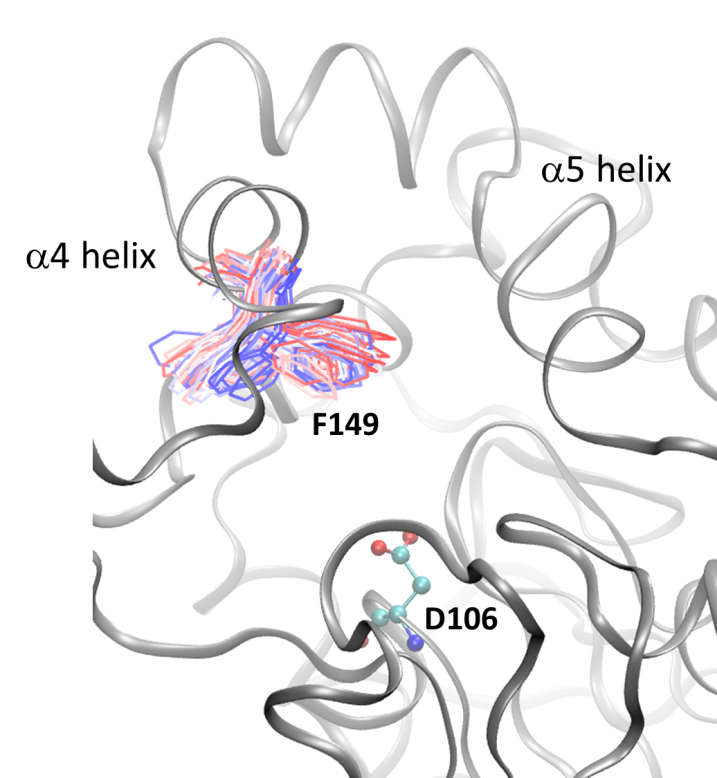


Supplementary Figure S15. Conformations of the F149 residue in closed and open states. Superimposition of representative snapshots from the clusters of DhaA31 with DCP at different states of the unbinding pathway presented in Figure 4B (main text). F149 is depicted as wires, coloured red for frames with DCP in the most bound state, and blue for frames with DCP in the most unbound state. The side chain of F149 can point towards α5 helix, blocking the tunnel (“closed”), or point towards the side, under α4 helix (“open”).

**A B**

**
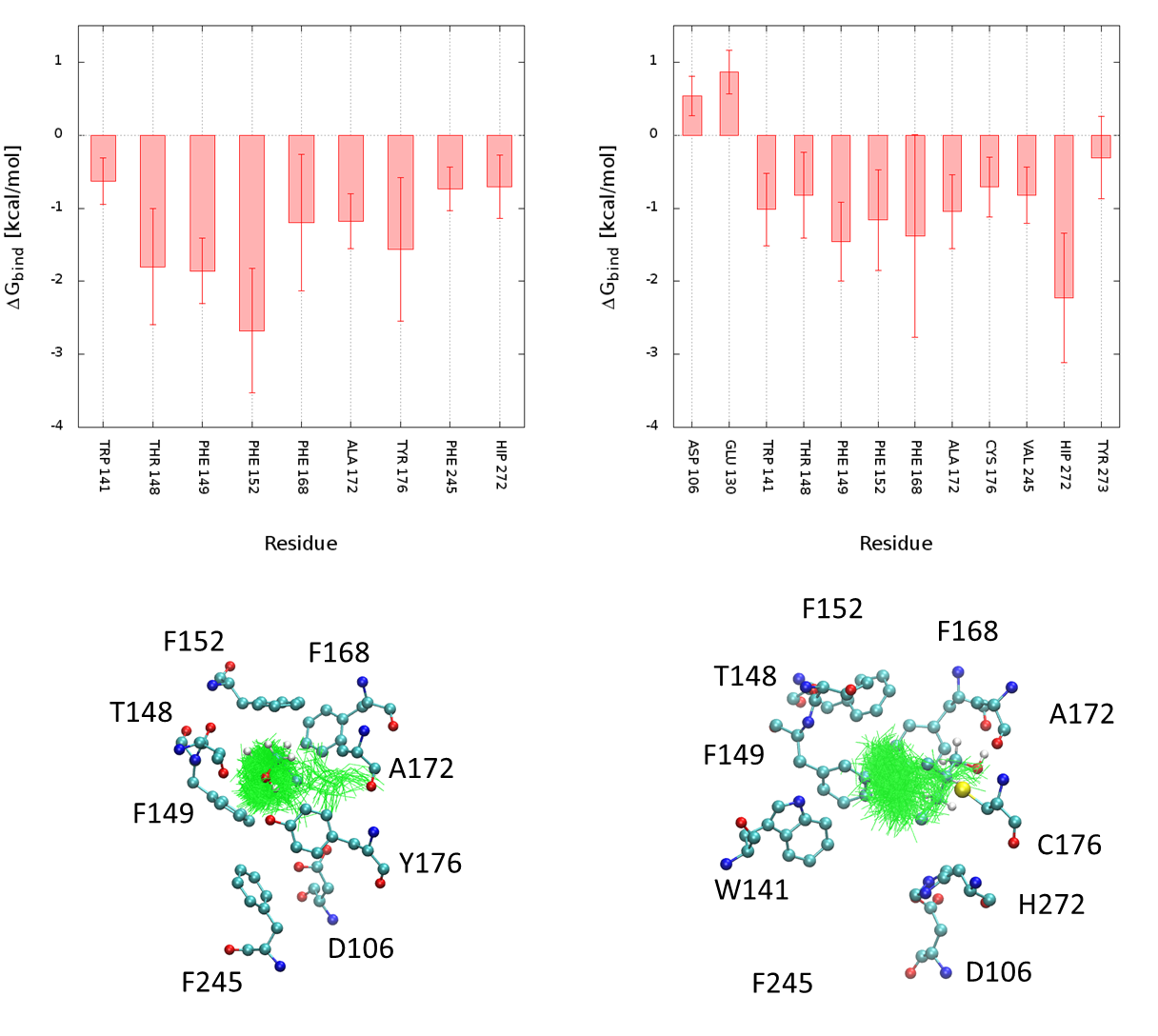
**

Supplementary Figure S16. Binding free energy of the protein residues with DCP at the global minimum, for DhaA31 (A) and DhaAwt (B). The average interactions are represented by the solid bars and the SD by the error bars (top row); the main residues are superimposed with the respective DCP cluster (bottom row). Only residues with ΔG or SD values ≥ 0.5 kcal/mol are displayed. The residues in DhaA31 interacting the most with DCP were F152 > F149 > T148 > Y176, and for DhaAwt it were H272 > F149 > F168 > F152 >> C176.

**A B**

**
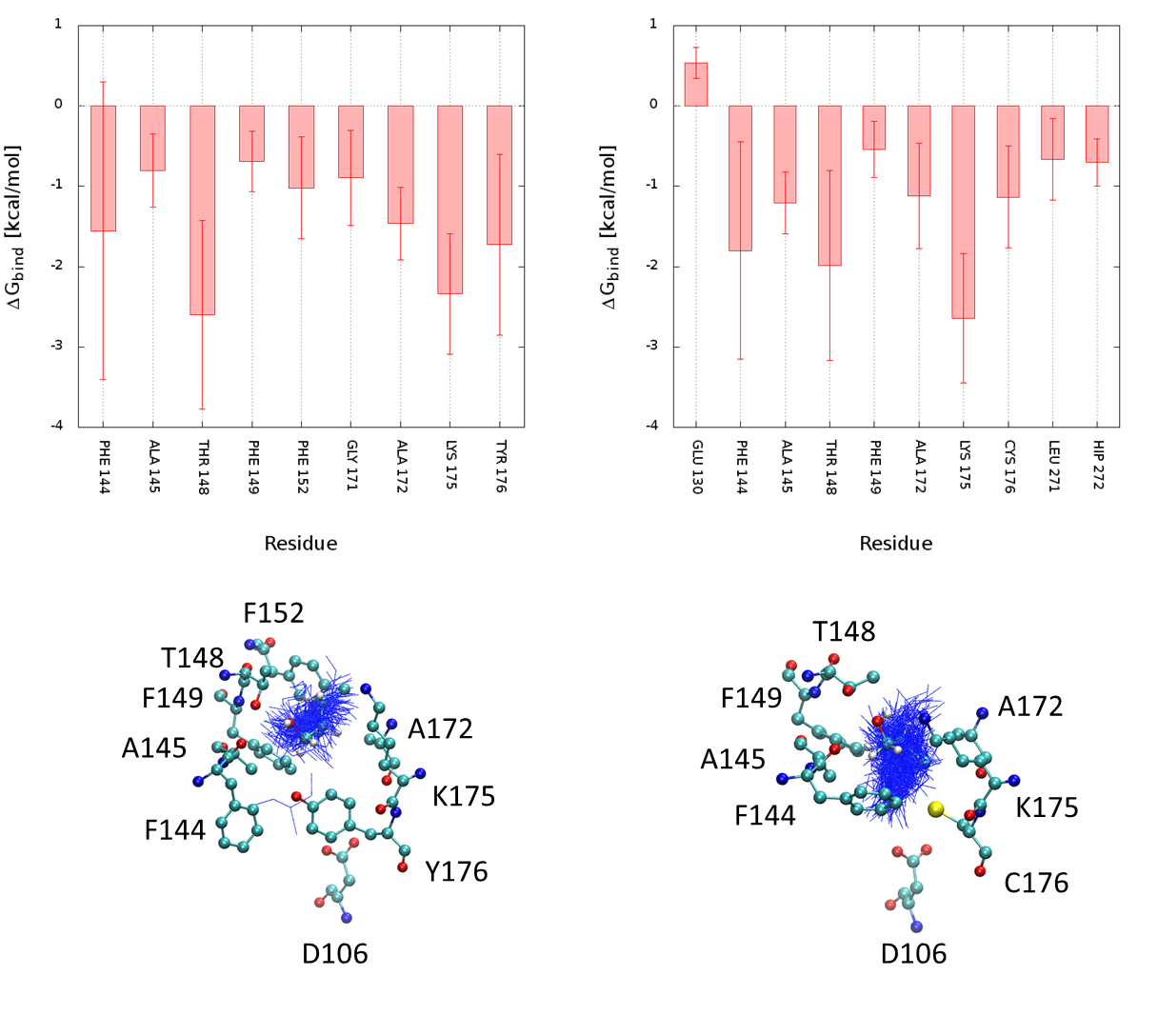
**

Supplementary Figure S17. Binding free energy of the protein residues with DCP at the TS1, for DhaA31 (A) and DhaAwt (B). The average interactions are represented by the solid bars and the SD by the error bars (top row); the main residues are superimposed with the respective DCP cluster (bottom row). Only residues with ΔG or SD values ≥ 0.5 kcal/mol are displayed. The dominant interactions in both enzymes were more shifted to the residues at the tunnel mouth, namely F144, T148, G171 and K175 in DhaA31, and F144, T148 and K175 in DhaAwt

**A B**


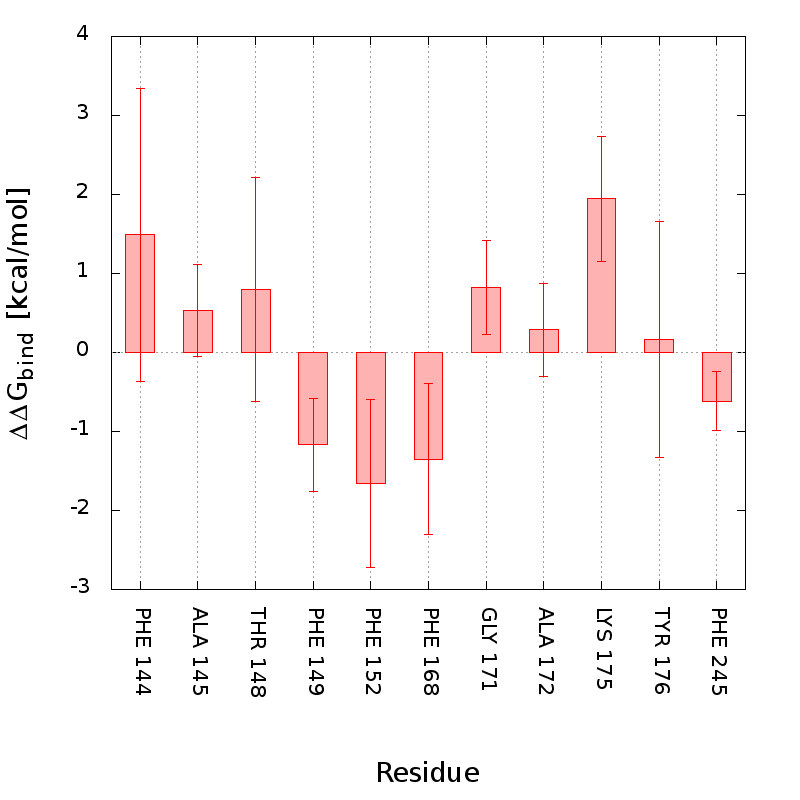

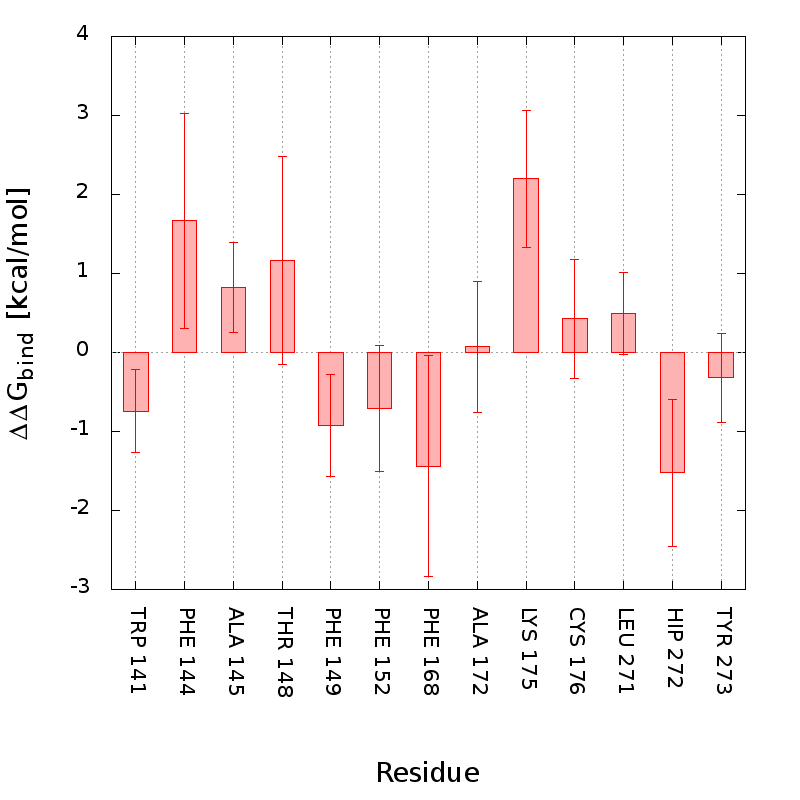


Supplementary Figure S18. Binding free energy difference between the global minimum and the TS1 states (ΔΔG = ΔG^min^ – ΔG^TS1^), for DhaA31 (A) and DhaAwt (B). The average interaction differences are represented by the solid bars and the SD by the error bars. Only residues with ΔΔG or SD values ≥ 0.5 kcal/mol are displayed.

***Supplementary Tables***

Supplementary Table S1. Residues lining the p1 tunnel selected for defining the path CV in DhaA31 and DhaAwt. The residues mutated in DaA31 are represented in bold italic.

| **Residue**  **Number** | **Residue name** | |
| --- | --- | --- |
|  | **DhaA31** | **DhaAwt** |
| 41 | ASN | ASN |
| 106 | ASP | ASP |
| 107 | TRP | TRP |
| 141 | TRP | TRP |
| 144 | PHE | PHE |
| 145 | ALA | ALA |
| 148 | THR | THR |
| 149 | PHE | PHE |
| 152 | PHE | PHE |
| 161 | LEU | LEU |
| 167 | ALA | ALA |
| 168 | PHE | PHE |
| 171 | GLY | GLY |
| 172 | ALA | ALA |
| 175 | LYS | LYS |
| ***176*** | ***TYR*** | ***CYS*** |
| 209 | LEU | LEU |
| ***245*** | ***PHE*** | ***VAL*** |
| 246 | ILE | LEU |
| 272 | HIP | HIP |
| ***273*** | ***PHE*** | ***TYR*** |
| - | DCP | DCP |

Supplementary Table S2. Kinetic parameters and statistics and obtained for the release of DCP form DhaA31 and DhaAwt.^a^

|  | **DhaA31** | **DhaAwt** |
| --- | --- | --- |
| **** [ns]** | 1.01 ± 0.97 × 10^8^ | 4.9 ± 2.2 × 10^3^ |
| ******_off_** [ns]** | 3.5 ± 1.4 × 10^4^ | 9.9 ± 5.6 × 10^2^ |
| ***p-value*** | 0.1968 | 0.1515 |
| ***k*_off_ [s^-1^]** | 2.8 ± 1.2 × 10^4^ | 1.01 ± 0.56 × 10^6^ |

^a^*μ*, mean release time; *τ*_off_, average dissociation time; *k*_off_, calculated dissociation rate; the variability of *μ* is the SEM, while for *τ*_off_ and *k*_off_ it is the SD obtained from a bootstrap analysis.

Supplementary Table S3. Relevant stages of the free energy surface for the unbinding of DCP from DhaA31 and DhaAwt.

| **DhaA31** | | | | **DhaAwt** | | | |
| --- | --- | --- | --- | --- | --- | --- | --- |
| ***d1***  **[Å]** | **Remark^a^** | **ΔG**  **[kcal/mol]** | **Ensemble size^b^** | ***d1***  **[Å]** | **Remark^a^** | **ΔG**  **[kcal/mol]** | **Ensemble size^b^** |
| ≤ 0.20 | 0 dist. | 7.76 | 59 | ≤ 0.10 | 0 dist. | 7.45 | 28 |
| 3.28 | shoul. | 1.65 | 546 | 3.46 | min. | 0.52 | 345 |
| 5.94 | glob. min. | 0.00 | 440 | 4.89 | glob. min. | 0.00 | 289 |
| 9.48 | max. – TS1 | 4.81 | 105 | 9.04 | max – TS1 | 2.26 | 215 |
| 12.14 | min. | 4.00 | 323 | 10.04 | min. | 1.92 | 323 |
| 14.00 | max. – TS2 | 4.61 | 106 | 11.83 | max. – TS2 | 3.38 | 77 |
| 15.62 | min. | 4.22 | 356 | 13.01 | min. | 2.86 | 182 |
| ≥ 20.0 | glob. max. | 38.9 | 144 | 15.18 | shoul. | 3.52 | 104 |
|  |  |  |  | ≥ 20.0 | glob. max. | 31.5 | 153 |

^a^“0 dist.” stands for 0-distance or minimum value of *d1*; “shoul.”, shoulder; “min.”, minimum; “max.”, maximum; “glob. min.”, global minimum; “glob. max.”, global maximum; TS1, transition state 1; TS2, transition state 2; ^b^size of the representative ensemble in number of snapshots in *d1* ± 0.001 nm.

Supplementary Table S4. Energy parameters from CaverDock calculations for the transport of DCP through the p1 tunnel of DhaA31 and DhaAwt.^a^

| **Calculation type** | **Protein** | **ΔE_min_** | **ΔE_max_** | **E_a_** | **Flexible residues** |
| --- | --- | --- | --- | --- | --- |
| **Rigid docking** | **DhaA31** | -4.1 | 5.8 | 9.9 | none |
|  | **DhaAwt** | -4.2 | -1.1 | 3.1 | none |
| **Flexible docking** | **DhaA31** | -4.1 | 0.2 | 4.3 | F149, Y176 |
|  | **DhaAwt** | -4.1 | -1.4 | 2.7 | F149, C176 |

^a^ΔE_min_, binding energy minimum at the bound state; ΔE_max_, binding energy maximum at the bound state; E_a_, activation energy barrier (E_a_ = ΔE_max_ - ΔE_min_). Binding energy is in kcal/mol.

*Supplementary Discussions*

Supplementary Discussion S1. Setting up the CV used in the MTD calculations.

We selected 9 snapshots containing DCP in different orientations and increasing the distance to the active site for both proteins. Several residues in contact with DCP were selected to be part of the path CV (Fig. S1 and Table S1 in Supplementary Material), to be used in the MTD simulations for describing the unbinding process. A first set of MTD simulations was run with the path CV only, but resulted in unsatisfactory Kolmogorov-Smirnoff statistics (*p-value* < 0.05). This was probably due to the path being insufficient to properly describe the system under study, as observed in other cases (Tiwary et al. 2015). To lift the possible degeneracy of the path CV, the ligand distance to the active site was added as a second variable in the set of CVs. The combination of path and distance CVs proved successful to achieve satisfactory *p-values* from the Kolmogorov-Smirnoff test of the unbinding (see below). A CV based on the distance alone was not tested here, but it proved unsuitable in other reported cases (Casasnovas et al. 2017).

Supplementary Discussion S2. The effects of the force field and solvent model in the calculation of kinetic rates.

The effects of the force field and solvent on the predicted kinetic rates were tested by additional adaptive sampling simulations performed with the ff14SB force field and OPC3 water model (Table 1). The ff12SB force field and TIP3P model were originally chosen because they were used on previous studies with the same systems (Marques et al. 2017). When the ff14SB force field was used, lower or similar *k*_off_ values were obtained, which may be attributed to different protein dynamics due to the force field. The ff14SB is expected to reproduce more accurately the experimental data for the backbone and side chains of the proteins (Maier et al. 2015; Zeng et al. 2016). A greater difference was observed with the OPC3 water model, which reduced further the unbinding rates. This was expected, since the TIP3P model is known to significantly overestimate the self-diffusivity of water and underestimate its viscosity. Conversely, the OPC3 is known to predict fairly well the bulk properties of liquid water, namely the dipole moment, density and self-diffusion coefficient (Izadi and Onufriev 2016), which might significantly influence the process of DCP unbinding. When compared to the available experimental data, the *K*_d_ value predicted for DhaAwt using the combination ff14SB+OPC3 (8.3 ± 2.5 mM) was the closest to the experimental value (*K*_d_ = 0.95 ± 0.34 mM). This seems to suggest that these conditions can better represent the physical properties of that system. Here we have demonstrated that the choice of the method, force field and water model can have a high impact on the prediction of kinetic properties. However, it has also been found that the combination of more recent force fields and solvent models does not necessarily bring more accuracy to the calculations, if there is an imbalance between the protein-water and protein-protein interactions (Zhang et al. 2018). On the other hand, the general difficulty in accurately predicting dissociation rates from MD simulations has been reported by others, and it may be also associated with the complexity of the unbinding processes (Sun et al. 2017; Callegari et al. 2017). In our particular case, we have seen that the conformational isomerization of the free DhaAwt regulates the binding/unbinding equilibrium of DCP, and this is not a unique case (Seo et al. 2014). This may introduce extra difficulty in the correct prediction of absolute kinetic rates. However, important conclusions can be inferred from the comparative study of the two systems, namely the higher propensity of DhaAwt to release DCP as compared to DhaA31. This strongly supports the value of comparative studies with similar systems, namely for the design of new enzyme variants in protein engineering.

*References*

Callegari, D., Lodola, A., Pala, D., Rivara, S., Mor, M., Rizzi, A., et al. (2017). Metadynamics Simulations Distinguish Short- and Long-Residence-Time Inhibitors of Cyclin-Dependent Kinase 8. *J. Chem. Inf. Model.* 57, 159–169. doi:10.1021/acs.jcim.6b00679.

Casasnovas, R., Limongelli, V., Tiwary, P., Carloni, P., and Parrinello, M. (2017). Unbinding Kinetics of a p38 MAP Kinase Type II Inhibitor from Metadynamics Simulations. *J. Am. Chem. Soc.* 139, 4780–4788. doi:10.1021/jacs.6b12950.

Izadi, S., and Onufriev, A. V. (2016). Accuracy limit of rigid 3-point water models. *The Journal of Chemical Physics* 145, 074501. doi:10.1063/1.4960175.

Maier, J. A., Martinez, C., Kasavajhala, K., Wickstrom, L., Hauser, K. E., and Simmerling, C. (2015). ff14SB: Improving the Accuracy of Protein Side Chain and Backbone Parameters from ff99SB. *J. Chem. Theory Comput.* 11, 3696–3713. doi:10.1021/acs.jctc.5b00255.

Marques, S. M., Dunajova, Z., Prokop, Z., Chaloupkova, R., Brezovsky, J., and Damborsky, J. (2017). Catalytic Cycle of Haloalkane Dehalogenases Toward Unnatural Substrates Explored by Computational Modeling. *J. Chem. Inf. Model.* 57, 1970–1989. doi:10.1021/acs.jcim.7b00070.

Seo, M.-H., Park, J., Kim, E., Hohng, S., and Kim, H.-S. (2014). Protein conformational dynamics dictate the binding affinity for a ligand. *Nat Commun* 5, 3724. doi:10.1038/ncomms4724.

Sun, H., Li, Y., Shen, M., Li, D., Kang, Y., and Hou, T. (2017). Characterizing Drug–Target Residence Time with Metadynamics: How To Achieve Dissociation Rate Efficiently without Losing Accuracy against Time-Consuming Approaches. *J. Chem. Inf. Model.* 57, 1895–1906. doi:10.1021/acs.jcim.7b00075.

Tiwary, P., Limongelli, V., Salvalaglio, M., and Parrinello, M. (2015). Kinetics of protein-ligand unbinding: Predicting pathways, rates, and rate-limiting steps. *Proc. Natl. Acad. Sci. U.S.A.* 112, E386-391. doi:10.1073/pnas.1424461112.

Zeng, J., Li, Y., Zhang, J. Z. H., and Mei, Y. (2016). Examination of the quality of various force fields and solvation models for the equilibrium simulations of GA88 and GB88. *J Mol Model* 22, 177. doi:10.1007/s00894-016-3027-8.

Zhang, H., Yin, C., Jiang, Y., and van der Spoel, D. (2018). Force Field Benchmark of Amino Acids: I. Hydration and Diffusion in Different Water Models. *J Chem Inf Model* 58, 1037–1052. doi:10.1021/acs.jcim.8b00026.
